# *Helicobacter pylori* Exploits a Unique Repertoire of Type IV Secretion System Components for Pilus Assembly at the Bacteria-Host Cell Interface

**DOI:** 10.1371/journal.ppat.1002237

**Published:** 2011-09-01

**Authors:** Carrie L. Shaffer, Jennifer A. Gaddy, John T. Loh, Elizabeth M. Johnson, Salisha Hill, Ewa E. Hennig, Mark S. McClain, W. Hayes McDonald, Timothy L. Cover

**Affiliations:** 1 Department of Microbiology and Immunology, Vanderbilt University School of Medicine, Nashville, Tennessee, United States of America; 2 Department of Medicine, Vanderbilt University School of Medicine, Nashville, Tennessee, United States of America; 3 Proteomics Laboratory, Mass Spectrometry Research Center, Vanderbilt University School of Medicine, Nashville, Tennessee, United States of America; 4 Department of Gastroenterology and Hepatology, Medical Center for Postgraduate Education, and Department of Oncological Genetics, Cancer Center Institute, Warsaw, Poland; 5 Veterans Affairs Tennessee Valley Healthcare System, Nashville, Tennessee, United States of America; Fred Hutchinson Cancer Research Center, United States of America

## Abstract

Colonization of the human stomach by *Helicobacter pylori* is an important risk factor for development of gastric cancer. The *H. pylori cag* pathogenicity island (*cag* PAI) encodes components of a type IV secretion system (T4SS) that translocates the bacterial oncoprotein CagA into gastric epithelial cells, and CagL is a specialized component of the *cag* T4SS that binds the host receptor α5β1 integrin. Here, we utilized a mass spectrometry-based approach to reveal co-purification of CagL, CagI (another integrin-binding protein), and CagH (a protein with weak sequence similarity to CagL). These three proteins are encoded by contiguous genes in the *cag* PAI, and are detectable on the bacterial surface. All three proteins are required for CagA translocation into host cells and *H. pylori*-induced IL-8 secretion by gastric epithelial cells; however, these proteins are not homologous to components of T4SSs in other bacterial species. Scanning electron microscopy analysis reveals that these proteins are involved in the formation of pili at the interface between *H. pylori* and gastric epithelial cells. Δ*cagI* and Δ*cagL* mutant strains fail to form pili, whereas a Δ*cagH* mutant strain exhibits a hyperpiliated phenotype and produces pili that are elongated and thickened compared to those of the wild-type strain. This suggests that pilus dimensions are regulated by CagH. A conserved C-terminal hexapeptide motif is present in CagH, CagI, and CagL. Deletion of these motifs results in abrogation of CagA translocation and IL-8 induction, and the C-terminal motifs of CagI and CagL are required for formation of pili. In summary, these results indicate that CagH, CagI, and CagL are components of a T4SS subassembly involved in pilus biogenesis, and highlight the important role played by unique constituents of the *H. pylori cag* T4SS.

## Author Summary

*Helicobacter pylori* persistently colonizes the stomach in approximately half of the human population. People who are infected with *H. pylori* strains harboring the *cag* pathogenicity island (PAI) have an increased risk of developing gastric cancer. The *cag* PAI encodes a type IV secretion system (T4SS) that is utilized by the bacteria to inject the bacterial oncoprotein CagA into gastric epithelial cells. Related T4SSs found in several other bacteria have been studied in detail, but thus far there has been very little study of the *H. pylori cag* T4SS. Here, we utilized a mass spectrometry-based approach to reveal co-purification of three constituents of the *H. pylori* T4SS (CagH, CagI, and CagL) that lack homology to components of T4SSs in other bacterial species. These proteins are essential for CagA translocation into host cells, and scanning electron microscope studies reveal that the proteins are involved in the formation of pili at the bacterial-host cell interface. A conserved C-terminal motif present in CagH, CagI, and CagL is essential for functionality of the T4SS. This study highlights the important role played by unique constituents of the *H. pylori cag* T4SS, and illustrates the marked variation that exists among bacterial T4SSs.

## Introduction

*Helicobacter pylori* infection is associated with a significantly increased risk for the development of several gastric diseases, including peptic ulceration, gastric adenocarcinoma, and gastric lymphoma [1]-[4]. *H. pylori* is a genetically diverse species, and the risk of developing these diseases is dependent in part on characteristics of the *H. pylori* strain with which an individual is infected. In particular, *H. pylori* strains harboring the *cag* pathogenicity island are associated with a higher rate of disease than are strains lacking this determinant [5]–[7].

The *cag* pathogenicity island is a 40-kilobase chromosomal region that is predicted to encode 27 proteins [8], [9]. One of these proteins, CagA, is an immunodominant antigen that enters gastric epithelial cells and causes numerous cellular alterations [10]–[13]. Additional proteins encoded by the *cag* PAI comprise a type IV secretion system (T4SS) that translocates CagA into gastric epithelial cells [11], [12], [14]–[17]. Following translocation, CagA is phosphorylated by host cell kinases at tyrosine residues contained within EPIYA motifs in the C-terminal region of the protein [10], [14], [18]. CagA, in both phosphorylated and non-phosphorylated forms, is able to interact with host cell signaling proteins, resulting in an assortment of consequences, including cytoskeletal alterations, disruption of cellular junctions, and altered cellular adhesion and polarity [10]–[12], [19].

When co-cultured with gastric epithelial cells, *H. pylori* strains containing the *cag* PAI stimulate gastric epithelial cells to synthesize and secrete proinflammatory cytokines, such as interleukin-8 (IL-8) [20], [21]. One mechanism leading to IL-8 induction involves entry of *H. pylori* peptidoglycan into the epithelial cell cytoplasm, where it is recognized by the pathogen recognition molecule NOD1; entry of peptidoglycan into the cytoplasm occurs through a *cag* PAI-dependent process [22]. A second mechanism leading to IL-8 induction involves translocation of CagA. The CagA protein can induce upregulation of IL-8 transcription via activation of the Ras/Raf signaling pathway [23]; upregulation of IL-8 transcription by CagA is detectable in some CagA-positive strains but not others [23]–[26].

T4SSs are utilized by a wide variety of bacterial species for delivery of effector proteins or DNA-protein complexes into an assortment of recipient cells, including mammalian cells, plant cells, fungi, or other bacteria [27]–[31]. Our current understanding of bacterial T4SSs is based in large part on elegant studies of the *Agrobacterium tumefaciens* T4SS, which translocates bacterial DNA into plant cells [28], [30]. Thus far there has been relatively little study of the *H. pylori cag* T4SS. Initial identification of bacterial genes required for function of the *H. pylori cag* T4SS was accomplished by systematic transposon mutagenesis of genes within the *cag* PAI [21], or by inserting antibiotic cassettes into selected genes within the *cag* PAI [8], [9], [32]. One study reported that 17 *cag* genes are required for translocation of CagA into gastric epithelial cells, and 14 *cag* genes influence the secretion of IL-8 [21]. Several of these genes encode products that exhibit low-level sequence similarity to components found in the T4SSs of *A. tumefaciens* and other bacterial species [11], [12], [14]. At least 9 of the *cag* genes that are reported to be required for function of the *cag* T4SS lack homologs in other bacterial species [12], [21], [33]. The functions of the encoded proteins remain largely uncharacterized. Structural analyses have been undertaken for several components of the *cag* T4SS [17], and several studies have shown that Cag proteins can interact to form subassemblies [33]–[36]. Overall, there is only a very limited understanding of the structural organization of the *cag* T4SS apparatus.

Surface-exposed pili are an important feature of T4SSs [28], [37]. The T4SS pili of *A. tumefaciens* are comprised of VirB2 (the major pilin subunit) and VirB5 (the minor pilin subunit) [28], [37]. When *H. pylori* is in contact with gastric epithelial cells, the bacteria express pili that are associated with the *cag* T4SS [38]-[41]. Relatively little is known about the composition and biogenesis of these *H. pylori* pili. In contrast to T4SS pili from other bacterial species, the *H. pylori* T4SS pili are reported to be sheathed organelles [38]. *H. pylori* CagC is reported to be a VirB2 homolog [40], but there is not yet convincing evidence that CagC is a major component of *H. pylori* pili. Several of the *H. pylori* proteins (CagY, CagT, and CagX) that have been localized to the pili by immunogold staining [38], [41] are distantly related to core complex components in other T4SS (VirB10, VirB7, and VirB9, respectively). *H. pylori* CagL has also been localized to pili by immunogold staining and it is suggested that it is a minor pilus component [39]; however, CagL and VirB5 do not exhibit any detectable sequence similarity. Taken together, these findings suggest that the pili associated with the *H. pylori cag* T4SS are considerably different from the T4SS pili of *A. tumefaciens* and other known bacterial T4SSs.

CagL is a 26 kDa protein component of the *cag* T4SS that lacks homologs in other bacterial species. In various studies, CagL has been detected as a constituent of the T4SS pilus [39], on the surface of *H. pylori* [42], or as a protein localized to the soluble fraction of *H. pylori* lysates [38], [43]. *H. pylori* mutants lacking *cagL* are defective in the ability to translocate CagA into host cells and do not stimulate production of IL-8 by gastric epithelial cells [21], [39]. An important feature of CagL is its ability to bind α5β1 integrin [39]. CagL contains a canonical integrin-binding RGD motif, but there is not uniform agreement about the functional significance of this RGD motif in mediating binding of *H. pylori* to α5β1 integrin [39], [44]. A recent study reported that recombinantly expressed CagL functionally mimics fibronectin in its ability to induce focal adhesion formation in mouse fibroblasts and stimulate spreading of AGS cells [42]. Another study reported that CagL mediates dissociation of ADAM17 from α5β1 integrin [45]. This leads to NFκB-mediated repression of the gastric H,K-adenosine triphosphate α-subunit (HKα), which may ultimately contribute to transient hypochlorhydria in *H. pylori*-infected individuals [45]. Recently it was reported that, in addition to CagL, three other *H. pylori* proteins encoded by the *cag* PAI (CagA, CagI, and CagY) can interact with β1 integrin [44]. Interactions of these Cag proteins with β1 integrin have been detected using several approaches, including yeast two-hybrid screening, analysis of interactions between these proteins and integrin on the surface of gastric epithelial cells, and surface plasmon resonance analysis [44].

Since CagL is an important component of the *cag* T4SS that interacts directly with epithelial cells, we reasoned that CagL might physically interact with other T4SS components, and that such interactions might be required for CagL export, localization, stability, or activity. In the current study, we report that two other proteins encoded by the *cag* PAI (CagH and CagI) co-purify with CagL, and we provide evidence that these three proteins are components of one or more subassemblies associated with the *cag* T4SS. CagL, CagI, and CagH are not homologous to components of T4SSs in other bacterial species, but we demonstrate that all three proteins are essential for activity of the *cag* T4SS. We show that all three proteins are detectable on the bacterial surface. Scanning electron microscopy studies reveal that CagI and CagL are required for formation of pili at the interface between *H. pylori* and gastric epithelial cells, and a Δ*cagH* mutant strain produces pili with a distorted morphology compared to the pili of wild-type bacteria. This suggests that CagH functions as a regulator of pilus dimensions. Furthermore, we show that CagH, CagI, and CagL proteins contain a conserved C-terminal hexapeptide motif that is critical for T4SS functionality, and in the case of CagI and CagL, this motif is required for T4SS pilus formation. These studies highlight the important functions of these unique *H. pylori cag* T4SS components and illustrate the marked variation that exists among bacterial T4SSs.

## Results

### Identification of Cag Proteins that Co-Purify with CagL

**Figure 1:**
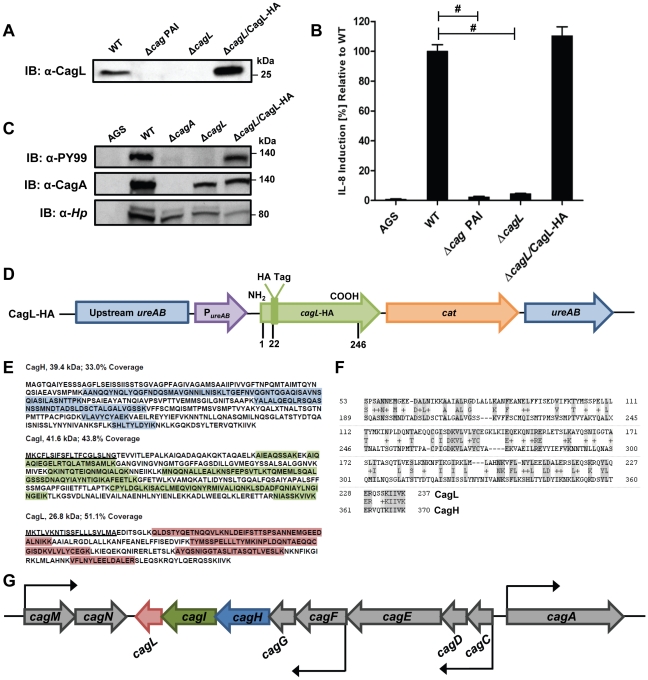
Functional analysis of CagL. **(A)** Immunoblot (IB) analysis indicates that CagL is expressed in wild-type *H. pylori* 26695 (WT), but not in Δ*cag* PAI or Δ*cagL* mutants. Complementation of Δ*cagL* with a gene encoding an epitope-tagged form of CagL (CagL-HA) results in restoration of CagL expression. **(B)** AGS cells were infected with WT *H. pylori* or the indicated mutants, and levels of IL-8 secreted by AGS cells were quantified by ELISA. A wild-type strain was able to induce IL-8 secretion, whereas mutants deficient in *cagL* were unable to induce IL-8 secretion. Complementation of CagL rescued the ability of Δ*cagL* to stimulate IL-8 secretion. Values from six replicate samples were compared to the wild-type control by ANOVA followed by Dunnett's *post hoc* test; # indicates p<0.05. **(C)** AGS cells were co-cultured with WT *H. pylori* or the indicated mutants, and tyrosine-phosphorylated CagA was detected using an anti-phosphotyrosine antibody (α-PY99). Samples also were immunoblotted with an anti-CagA antibody and antiserum reactive with multiple *H. pylori* proteins (α-*Hp*). The Δ*cagL* mutant was defective in ability to translocate CagA into AGS cells. **(D)** Schematic of a construct encoding an epitope-tagged form of CagL (CagL-HA), used to complement Δ*cagL* in *cis* from the *ureA* locus. P*_ure_*_AB_, *ure*AB promoter; *cat*, chloramphenicol resistance mediated by chloramphenicol acetyltransferase gene of *C. coli*. **(E)** CagH and CagI were each co-immunoaffinity purified with the target protein CagL from WT lysate. CagH, CagI, and CagL peptides identified by MudPIT (pooled data from all CagL purifications shown in Table 1 and 2) are indicated. N-terminal signal peptides (underlined) are predicted to be present in CagI and CagL, based on SignalP analysis [82], [83]. **(F)** An alignment of CagH and CagL protein sequences reveals sequence similarity between the C-terminal portion of CagH and the corresponding portion of CagL (32% amino acid identity, 47% similarity in the region shown). **(G)** Operon structure of the *cag* PAI region spanning from *cagA* to *cagM* [46]. Black arrows indicate transcriptional start points. *cagH, cagI*, and *cagL* are contiguous genes within an operon spanning from *cagC* to *cagL* and a suboperon spanning from *cagF* to *cagL* [46].

Previous studies have shown that CagL is an important component of the *H. pylori cag* T4SS that can bind α5β1 integrin [21], [39], [44]. We hypothesized that CagL interacts with other components of the *cag* T4SS to form one or more subassemblies. Therefore, we sought to identify *H. pylori* proteins that co-purify with CagL. To facilitate these studies, a *cagL*-deficient *H. pylori* mutant strain (Δ*cagL*) was generated as described in Methods. As expected, CagL expression was detected by immunoblot analysis in the wild-type (WT) strain but not in the Δ*cagL* mutant strain (Figure 1A). Consistent with previous reports [21], [39], [44], the Δ*cagL* mutant strain was defective in the ability to stimulate IL-8 secretion by gastric epithelial cells (Figure 1B) and defective in the ability to translocate CagA into gastric epithelial cells (Figure 1C). Complementation with an epitope-tagged form of CagL restored the ability of the bacteria to translocate CagA into host cells and induce IL-8 secretion (Figure 1A–D).

We used a polyclonal anti-CagL serum to immunoaffinity-purify CagL from a wild-type *H. pylori* strain, and a Δ*cagL* mutant strain was processed in parallel. As expected, immunoblotting studies showed that CagL was immunoaffinity-purified from WT lysate but not Δ*cagL* lysate (data not shown). The protein content of each sample was analyzed using multidimensional protein identification technology (MudPIT). Numerous proteins were detected in each sample, but we focused in particular on proteins encoded by the *cag* PAI, since many of these are known or predicted to comprise components of the *cag* T4SS. CagL was identified by MudPIT in the preparation derived from WT lysate, but was not detected in the preparation derived from Δ*cagL* lysate (Table 1). In addition, the relative abundance of CagH and CagI peptides detected in affinity-purified preparations derived from the WT strain was significantly higher than the number of CagH and CagI peptides detected in preparations derived from the Δ*cagL* mutant strain (Table 1). Peptides identified for CagH, CagI, and CagL covered 33% to 51% of the respective proteins (Figure 1E). CagH and CagI co-purified with CagL from the WT strain in six independent experiments (data not shown). CagA and several other Cag proteins were also detected in the affinity purified preparations, but the numbers of spectral counts corresponding to these proteins were not significantly different when comparing preparations derived from WT and Δ*cagL* mutant strains (Table 1). Many other Cag proteins were detected by mass spectrometric analysis of crude bacterial lysate (Table S1), but were not detected in the affinity purified preparations. These initial experimental results, demonstrating co-purification of CagL, CagH, and CagI, suggested that these three proteins are components of one or more subassemblies associated with the T4SS.

**Table 1:** Cag proteins that co-purify with CagL.

		α-CagL^b^			
Gene Number a	Protein	WT	Δ*cagL*	WT	Δ*cagL*
HP0539	CagL	19 **	0	21 ^***^	0
HP0540	CagI	33 ^***^	0	29 ^***^	0
HP0541	CagH	17 **	0	19 ^***^	0
HP0547	CagA	33	13	32	27
HP0522	Cag3	2	0	0	0
HP0527	CagY	4	1	3	3
Total Spectral Counts		3009	1054	2829	2609

a Based on the *H. pylori* 26695 genome annotation.

b CagL was affinity purified from the WT strain (cultured for 48 h) using anti-CagL polyclonal antiserum, and a Δ*cagL* mutant was processed in parallel as a control. The Table shows numbers of spectral counts observed by MudPIT analysis for each identified Cag protein. The Table shows results from two independent experiments.

**p<0.01; *** p<0.001 when comparing WT with the Δ*cagL* mutant, according to the G-test likelihood ratio, post-spectral count normalization.

As another approach for identifying proteins that co-purify with CagL, we undertook experiments with a complemented Δ*cagL* mutant strain expressing CagL with an N-terminal hemagglutinin (HA) epitope tag (Figure 1D). We immunoaffinity-purified CagL-HA from lysate of the strain expressing CagL-HA, using an immobilized monoclonal anti-HA antibody; the WT strain (expressing CagL without an HA tag) was processed in parallel in the same manner. CagL-HA and co-purifying proteins were eluted from the anti-HA affinity column by HA peptide competition, and MudPIT was utilized to analyze the total protein content of the samples. Mass spectrometry identified only three Cag proteins in the sample purified from the strain expressing CagL-HA: the target protein CagL-HA, CagI, and CagH (Table 2). The relative abundance of CagL and CagI peptides detected in the preparation from the CagL-HA-expressing strain was significantly higher than the number of CagL and CagI peptides detected in the preparation from the WT strain. Of note, compared to affinity-purified preparations of CagL obtained using polyclonal antiserum, the preparation of CagL-HA obtained using a monoclonal antibody and peptide elution had a much higher level of purity (Table 2 and Table S2).

**Table 2:** Cag proteins that co-purify with epitope-tagged CagH, CagI, and CagL.

		α-HA b			α-FLAG c	
Gene Number a	Protein	WT	CagH-HA	CagL-HA	WT	CagI-FLAG
HP0540	CagI	0	203 ***	10 ^***^	0	170 ^***^
HP0541	CagH	1	91 ***	2	0	19 ^***^
HP0539	CagL	0	98 ***	21 ***	0	19 ^***^
HP0547	CagA	0	2	0	47	87
HP0544	CagE	0	0	0	6*	1
HP0535	CagQ	1	4	0	0	0
HP0530	CagV	0	0	0	4	4
HP0543	CagF	0	0	0	2	2
HP0528	CagX	0	0	0	2	6
HP0527	CagY	0	0	0	5	4
HP0520	Cag1	0	0	0	3	2
Total Spectral Counts		208	1428	237	6310	10942

a Based on the *H. pylori* 26695 genome annotation.

b CagH-HA or CagL-HA were affinity purified from strains expressing these proteins using an anti-HA antibody, and a WT strain was processed in parallel as a control. The Table shows numbers of spectral counts observed by MudPIT analysis for each identified Cag protein.

c CagI-FLAG was affinity purified from a strain expressing this protein using an anti-FLAG antibody, and a WT strain was processed in parallel as a control. The Table shows numbers of spectral counts observed by MudPIT analysis for each identified Cag protein.

*p<0.05; *** p<0.001 when compared to WT control, according to the G-test likelihood ratio, post-spectral count normalization.

**Figure 2:**
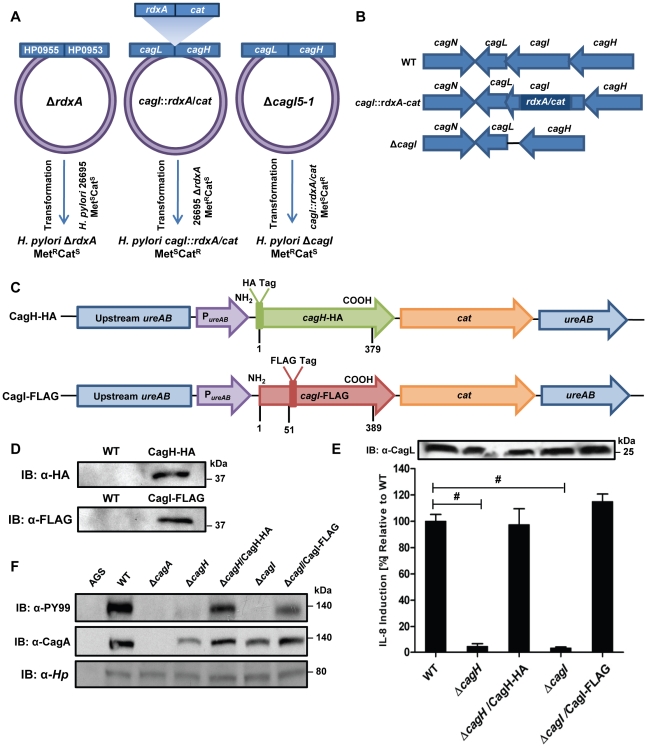
Functional analysis of CagH and CagI. **(A)** A metronidazole-resistant (Met^R^) strain of *H. pylori* was generated by transforming the WT strain with pMM672 (p-Δ*rdxA*); metronidazole-resistant transformants contained a deletion of the *rdxA* locus. The *cagI* chromosomal locus was deleted by transforming the metronidazole-resistant *H. pylori* with p-*cagI*::*rdxA*/*cat;* this resulted in chloramphenicol-resistant (Cat^R^), metronidazole-sensitive (Met^S^) colonies, designated *cagI*::*rdxA*/*cat*. To generate a mutant containing an unmarked *cagI* deletion, *H. pylori cag*I::*rdx*A/*cat* was transformed with pΔ*cagI*5-1, and metronidazole-resistant colonies were selected. **(B)** Schematic illustration of the chromosomal *cagH-cagI-cagL* region in the WT strain, *cagI::rdxA-cat* mutant, and Δ*cagI* unmarked mutant. A similar approach was used to generate an unmarked Δ*cagH* mutant strain. **(C)** Schematic of plasmids encoding epitope-tagged forms of CagH and CagI. P*_ure_*_AB_, *ure*AB promoter; *cat*, chloramphenicol resistance mediated by chloramphenicol acetyltransferase gene of *C. coli*. The hemagglutinin (HA) epitope sequence was inserted into CagH at the N-terminus (CagH-HA), and the FLAG epitope was inserted at residue 51 of CagI (CagI-FLAG). Numbers indicate position of residues in the epitope tagged proteins. These plasmids were introduced into Δ*cagH* and Δ*cagI* mutants, respectively, and the resulting complemented strains contained sequences encoding CagH-HA or CagI-FLAG, inserted into the *ureA* locus. **(D)** Immunoblot analysis of CagH-HA and CagI-FLAG in the complemented Δ*cagH* and Δ*cagI* mutant strains, using monoclonal antibodies directed against the epitope tags. **(E)** WT *H. pylori* or the indicated mutants were co-cultured with AGS cells for 4.5 h, and IL-8 expression was analyzed by ELISA. Δ*cagH* and Δ*cagI* mutants were defective in ability to induce IL-8 secretion, and complementation corrected the defect. Values from six replicate samples were compared to the wild-type control by ANOVA followed by Dunnett's *post hoc* correction; # indicates p<0.05. CagL remained detectable in all strains throughout the assay, as demonstrated by anti-CagL immunoblot (inset). **(F)** AGS cells were co-cultured with WT *H. pylori* or the indicated mutants, and tyrosine-phosphorylated CagA was detected with an anti-phosphotyrosine antibody (α-PY99). Samples also were immunoblotted with an anti-CagA antibody and antiserum reactive with multiple *H. pylori* proteins (α-*Hp*). CagA translocation was impaired in the Δ*cagH* and Δ*cagI* mutants.

To further investigate potential interactions among CagH, CagI, and CagL, we attempted to generate polyclonal antisera against CagH and CagI, but this failed to yield antisera that recognized the corresponding *H. pylori* proteins. Therefore, we generated unmarked non-polar Δ*cagH* and Δ*cagI* mutants (Figure 2A and B), as well as complemented mutant strains expressing epitope-tagged forms of CagH or CagI in a heterologous chromosomal locus (Figure 2C). CagH-HA and CagI-FLAG proteins of the expected masses were detected in the complemented bacteria (but not WT bacteria) by immunoblotting using monoclonal antibodies against the epitope tags (Figure 2D). We immunoaffinity-purified CagH-HA from lysate of the strain expressing this protein, and lysate from the WT strain (expressing CagH without an HA tag) was processed in parallel. The relative abundance of CagH, CagI and CagL detected in the preparation from the CagH-HA-expressing strain was significantly higher than the number of CagH, CagI and CagL peptides detected in the preparation from the WT strain (Table 2 and Table S2).

In a similar manner, we purified CagI-FLAG from the strain expressing this epitope-tagged protein. The relative abundance of CagI, CagH, and CagL detected in the preparation from the CagI-FLAG-expressing strain was significantly higher than the number of corresponding peptides detected in the preparation from the WT strain (Table 2). These experiments recapitulated the co-purification of CagH, CagI, and CagL that was observed in initial purifications utilizing polyclonal anti-CagL antisera (Table 1). In individual experiments, we sometimes detected levels of other proteins besides CagH, CagI, and CagL that were significantly increased in affinity-purified preparations compared to control preparations (Table S2), but these results were not consistently reproduced and were not recapitulated when alternate members of a putative CagH-CagI-CagL complex were targeted for purification.

Further analysis revealed that there is weak sequence similarity between the amino acid sequences of CagL and CagH (32% amino acid identity and 47% similarity in the C-terminal region, Figure 1F). CagL, CagI, and CagH are encoded by contiguous, overlapping genes in an operon [46] (Figure 1G). Other genes included in this operon, upstream from *cagH, cagI*, and *cagL*, include *cagC* (encoding the putative T4SS major pilin component [40]), *cagE* (homologous to *virB4*, encoding an ATPase required for T4SS function), and *cagF* (encoding a chaperone protein that binds to CagA) [47]. Since we observed highly reproducible co-purification of CagH, CagI, and CagL, and because genes within an operon often have related functions, we proceeded to undertake further studies of all three proteins.

### CagL Stability is Impaired in the Absence of CagH and CagI

**Figure 3:**
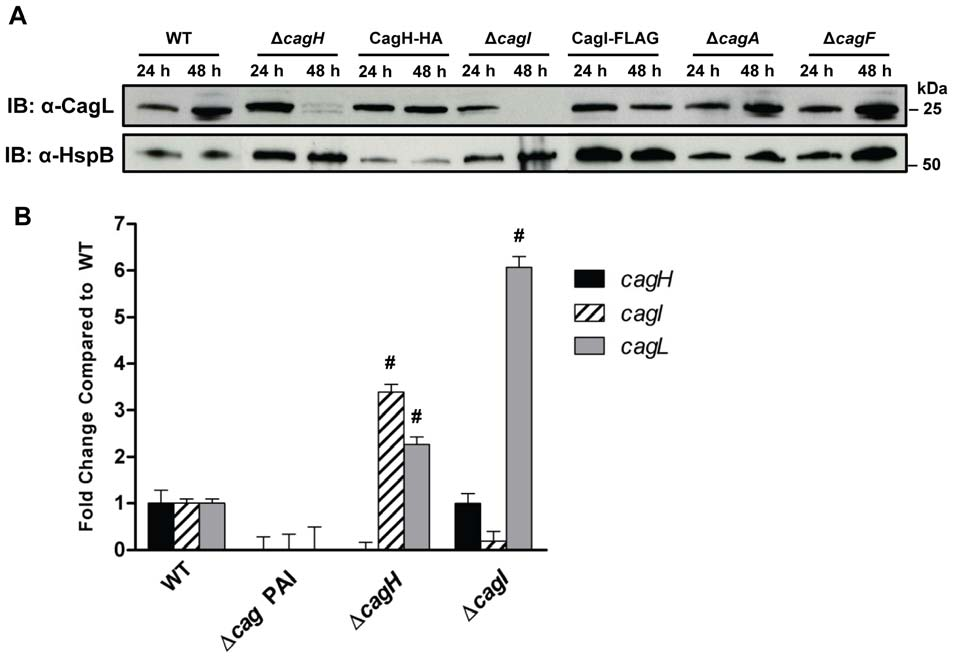
Analysis of CagL expression in Δ*cagH* and Δ*cagI* mutants. **(A)** *H. pylori* strains were cultured for 24 or 48 hours on solid media prior to lysis and immunoblotting to detect expression of CagL. Expression of HspB, a heat shock protein homolog, was analyzed as a control. In the Δ*cagH* and Δ*cagI* mutants, levels of CagL were markedly lower at the 48 h timepoint than at the 24 h timepoint. CagL levels remained stable at both timepoints in the WT strain and the complemented mutants expressing CagH-HA or CagI-FLAG. CagL stability is unaffected by absence of Cag proteins that do not interact with CagL (Δ*cagA* and Δ*cagF*). **(B)** RT-PCR analysis of *cagH*, *cagI*, and *cagL* transcription in the indicated WT and mutant bacteria, cultured for 48 h. *cagL* transcript levels are upregulated in the Δ*cagH* and Δ*cagI* mutants, compared to levels detected in the WT strain. # indicates p<0.05 according to one-way ANOVA with Dunnett's *post hoc* correction.

Previous studies have reported decreased stability of T4SS components if one or more interacting proteins are absent [36], [37], [43], [48], [49]. Based on the observed co-purification of CagI and CagH with CagL, we hypothesized that CagL stability might be impaired in the absence of CagI or CagH. To assess CagL protein stability, we analyzed bacterial lysates prepared from the WT strain, Δ*cagI* mutant, and Δ*cagH* mutant, each cultured for either 24 h or 48 h. CagL could be detected by immunoblotting of lysates from all three strains at 24 h of growth (Figure 3A). However, after 48 h of growth, CagL levels were markedly reduced in lysates from the Δ*cagI* and Δ*cagH* mutants compared to levels in WT lysate. CagL stability at the 48 h timepoint was increased by complementation of the mutant strains (Figure 3A). We also evaluated the stability of CagL in the absence of two Cag proteins selected as controls (CagA and CagF). We did not detect significant co-purification of CagA or CagF with CagL in the previous experiments (Table 1), and neither CagA nor CagF is required for functionality of the T4SS, based on analysis of IL-8 induction [21]. Absence of CagA or CagF did not have any detectable effect on CagL stability during early or late phases of growth (Figure 3A).

We next investigated the possibility that the observed reductions in CagL protein expression in Δ*cagH* and Δ*cagI* mutants might be attributable to a reduction in *cagL* transcription. Analysis of *cagL* transcription in each of these strains by real-time PCR indicated that *cagL* transcription was not diminished, but was in fact increased in the Δ*cagH* and Δ*cagI* mutants compared to the WT strain at 48 h of growth (Figure 3B). Thus, the observed reduction in CagL levels in the Δ*cagH* and Δ*cagI* mutant strains is attributed to reduced stability of CagL, rather than decreased transcription of *cagL*.

To further investigate potential relationships among CagL, CagI, and CagH, we analyzed whether co-purification of CagI with CagL was dependent on the presence of CagH. We used polyclonal anti-CagL antiserum to immunoaffinity purify CagL from both the WT strain and a Δ*cag*H mutant strain, each cultured for 24 h (which permitted stable expression of CagL in both strains). The samples were then analyzed by mass spectrometry. As expected, CagL was immunoaffinity purified from both the WT strain and the Δ*cagH* mutant (Table S3). CagI was co-purified along with CagL from the WT strain, but not from the Δ*cagH* mutant (Table S3). In a similar manner, we investigated whether co-purification of CagH with CagL was dependent on the presence of CagI. CagH was co-purified with CagL from both the WT strain and Δ*cagI* mutant; however, significantly lower levels of CagH were co-purified from the Δ*cagI* mutant strain (Table S3).

In summary, these experiments indicate that CagL stability is reduced in the absence of CagH or CagI, and co-purification of CagH or CagI with CagL is dependent on the presence of all three proteins. Taken together with the previous results (Table 1 and 2), these data provide evidence that CagL, CagI, and CagH are components of one or more subassemblies associated with the *cag* T4SS.

### Functional Properties of CagH and CagI

A previous study, which analyzed mutant strains containing transposon insertions in individual *cag* genes, provided an important foundation for identifying genes in the *cag* PAI that are required for functionality of the *H. pylori cag* T4SS [21]. This study reported that both CagH and CagI were required for CagA translocation, and that CagH (but not CagI) was required for IL-8 induction [21]. A limitation of the methodologic approach was that unrecognized spontaneous secondary mutations or polar effects associated with transposon insertion into the *cag* PAI could not be confidently excluded. To determine definitively whether CagH and CagI were required for the function of the *cag* T4SS, we analyzed the unmarked Δ*cagH* and Δ*cagI* deletion mutant strains and complemented mutants described in the previous section.

In comparison to the WT strain, both the Δ*cagH* and Δ*cagI* mutants were defective in their ability to induce IL-8 secretion in gastric epithelial cells (Figure 2E). Complementation of these mutants with genes encoding CagH-HA and CagI-FLAG, respectively, rescued the ability of the Δ*cagH* and Δ*cagI* mutants to induce IL-8 secretion at WT levels (Figure 2E). Immunoblot analysis indicated that CagL remained intact in the mutant strains throughout the assay (Figure 2E), which indicates that the inability of these mutant strains to induce IL-8 expression is attributable to absence of either CagH or CagI, respectively, rather than instability of CagL. The Δ*cagH* and Δ*cagI* mutants were also defective in their ability to translocate CagA into gastric epithelial cells (Figure 2F). The ability of these mutants to translocate CagA at levels comparable to WT was rescued by complementation with epitope-tagged versions of the corresponding proteins (Figure 2F). These results indicate that CagH and CagI, similar to CagL, are required for proper function of the *cag* T4SS.

### Localization of CagH, CagI, and CagL

**Figure 4:**
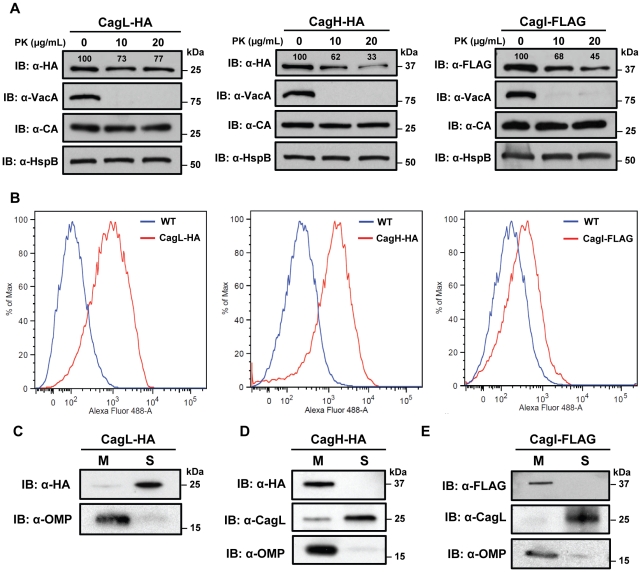
Localization of CagH and CagI. **(A)** Whole bacteria expressing CagL-HA, CagH-HA, or CagI-FLAG were harvested after 24 h of growth and subjected to proteolytic digestion with Proteinase K (PK). Susceptibility to cleavage by Proteinase K was monitored by immunoblotting using antibodies against the appropriate epitope tags. As a control, cleavage of VacA (a surface-exposed protein) and carbonic anhydrase (CA, an inner membrane protein with a periplasmic domain) was monitored. Heat shock protein (HspB) served as a loading control, and densitometry was performed as described in Methods. Numbers above bands representing CagL-HA, CagH-HA, and CagI-FLAG indicate the percent of protein remaining after cleavage by PK. **(B)** Flow cytometry analysis indicates that CagL-HA, CagH-HA, and CagI-FLAG proteins are detectable on the bacterial surface. **(C)** Bacteria expressing epitope-tagged CagL (CagL-HA), **(D)** CagH (CagH-HA), or **(E)** CagI (CagI-FLAG) were fractionated into total membrane (M) and soluble (S) fractions as described in Methods. CagL-HA, CagH-HA, CagI-FLAG, and untagged CagL were detected by immunoblotting. As a control, fractions were immunoblotted with a monoclonal antibody that recognizes an *H. pylori* outer membrane protein (α-OMP) (Santa Cruz).

**Figure 5:**
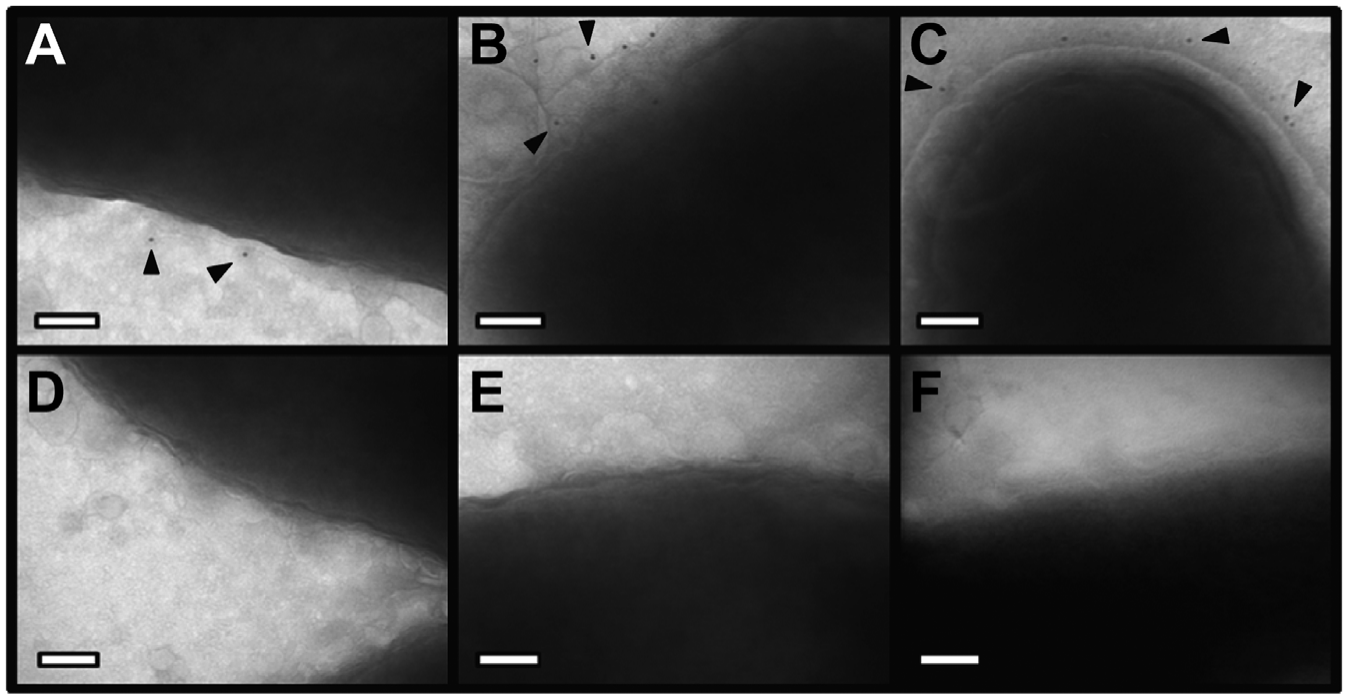
Immunoelectron microscopy analysis of whole bacterial cells. *H. pylori* strains were grown on blood agar plates and immunogold labeling was performed as described in Methods. **(A)** Δ*cagH*/CagH-HA, stained with anti-HA and gold-labeled secondary antibody. **(B)** Δ*cagI*/CagI-FLAG, stained with anti-FLAG and gold-labeled secondary antibody. **(C)** Δ*cagL*/CagL-HA, stained with anti-HA and gold-labeled secondary antibody. **(D)** WT, stained with anti-HA and gold-labeled secondary antibody. **(E)** WT, stained with anti-FLAG and gold-labeled secondary antibody. **(F)** WT, stained with secondary antibody only (representative image for all strains when treated with secondary antibody only). Magnification bars indicate 100 nm. Arrowheads indicate location of gold-labeled secondary antibody.

Since expression of CagH and CagI proteins in *H. pylori* has not been detected previously, we sought to analyze the subcellular localization of these proteins. As a first approach, we investigated potential surface exposure of CagH, CagI, and CagL by analyzing the susceptibility of these proteins, as well as several controls, to digestion by proteinase K. As expected, incubation of the intact bacteria with proteinase K was sufficient to cleave VacA, a protein known to be localized on the bacterial surface [50], [51] (Figure 4A). In contrast, carbonic anhydrase (CA), a protein known to have a periplasmic domain [52], was not susceptible to cleavage by proteinase K. Similarly, CagV (a VirB8 homolog), which is predicted to have domains in the periplasmic space [16], [17], [43], was also resistant to cleavage by proteinase K (data now shown). CagH-HA, CagI-FLAG, and CagL-HA were each susceptible to cleavage by proteinase K (Figure 4A), but in contrast to VacA, these three Cag proteins were incompletely digested. This suggests that CagH, CagI, and CagL are present on the bacterial surface, and in addition, another pool of these proteins exists in a site that is not susceptible to the protease. Consistent with the susceptibility of CagH-HA, CagI-FLAG, and CagL-HA to cleavage by proteinase K, surface exposure of all three proteins was detected by flow cytometry analysis (Figure 4B). Immunogold labeling studies also revealed localization of CagH-HA, CagI-FLAG, and CagL-HA on the surface of *H. pylori* (Figure 5). Typically fewer than 10 gold particles per bacterial cell were visualized on the peripheral margins of bacteria, which suggests either that the number of CagH, CagI, and CagL proteins on the bacterial surface under these conditions is relatively small, or that there may be limitations with the use of these monoclonal antibodies for immunogold labeling.

As another approach for analyzing the subcellular localization of CagH, CagI, and CagL, *H. pylori* strains expressing CagH-HA, CagI-FLAG, or CagL-HA were sonicated and fractionated in the absence of detergent. Soluble fractions (which are expected to contain cytoplasmic and periplasmic proteins) and total membrane fractions were analyzed by immunoblotting using monoclonal antibodies directed against the appropriate epitope tags. In agreement with a previous report [38], [43], the native form of CagL was localized mainly in the soluble fraction, and lesser amounts were present in the membrane fraction; similar results were observed for CagL-HA (Figure 4C, D, E). Both CagH-HA and CagI-FLAG were localized exclusively in the membrane fraction (Figure 4D, E). The insolubility of CagH and CagI in this experiment compared to previous experiments is attributed to the absence of detergent in these fractionated samples and the presence of detergent in earlier samples. We propose that, when complexed together, CagL is loosely tethered to membrane-associated CagH and CagI, and that in the absence of detergent, the shear forces or heat generated by sonication are sufficient to disrupt this interaction and solubilize CagL, but not CagH and CagI. An additional possibility is that the CagH-CagI-CagL complexes include the relatively small portion of membrane-associated CagL.

In summary, these experiments provide evidence that CagL, CagH, and CagI exist in multiple subcellular locations, including the surface of *H. pylori.* The existence of these proteins in multiple subcellular locations potentially reflects multiple stages of T4SS assembly. Localization of CagL mainly to the soluble fraction as well as on the bacterial surface helps to reconcile apparent contradictions in previous publications, which reported CagL localization to either the soluble fraction or surface-exposed sites [38], [39], [42], [43]. In agreement with a previous study [43], we propose that CagL exists mainly in a periplasmic pool, but it can also be associated with the outer membrane, perhaps loosely tethered to the cell surface. Distribution of at least two other Cag proteins (Cag3 and CagY) into multiple pools with different localizations has been suggested previously [33], [38]. Complexes comprised of CagH, CagI, and CagL could potentially exist in multiple sites, including the bacterial surface and the periplasm (where periplasmic CagL may interact with membrane-associated CagH and CagI proteins).

### CagH, CagI, and CagL have Key Roles in T4SS Pilus Formation

**Figure 6:**
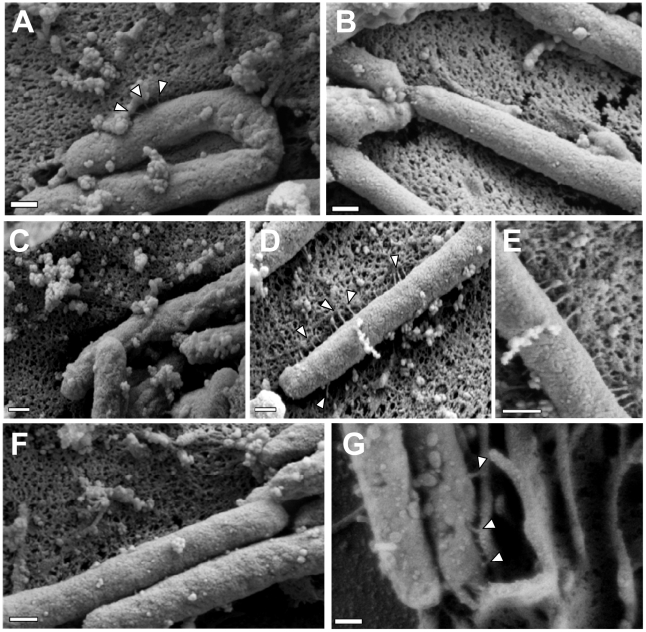
Role of CagI and CagL in pilus formation. WT *H. pylori*, mutant strains, and complemented mutant strains were co-cultured with AGS cells and then analyzed by FESEM. **(A)** WT. **(B)** Δ*cag* PAI. **(C)** Δ*cagL*. **(D)** Δ*cagL*/CagL-HA. **(E)** High magnification of pilus structures produced by Δ*cagL*/CagL-HA (panel D). **(F)** Δ*cagI*. **(G)** Δ*cagI/*CagI. Arrowheads indicate T4SS pilus structures. Magnification bars indicate 200 nm.

To investigate a possible mechanism by which CagH, CagI, and CagL might contribute to activity of the *cag* T4SS, we used scanning electron microscopy (FESEM) to analyze interactions of *H. pylori* with AGS gastric epithelial cells. Specifically, we tested the hypothesis that CagH, CagI, or CagL might be required for formation of pili at the bacterial-host cell interface. As expected, we detected pili at the interface between WT *H. pylori* and AGS cells (Figure 6A), and consistent with previous reports [38], [39], [41], these pili were not visualized when a Δ*cag* PAI mutant (Figure 6B), *cagT* (*virB7* homolog) mutant, or *cagE* mutant (data not shown) was co-cultured with AGS cells. Adherent WT bacteria exhibited pili on surfaces that were contiguous to the epithelial cells, and we did not observe pili on non-adherent bacteria. The dimensions of the pili were approximately 14 nm in width and 65 nm in length (Table 3), and the pili were distributed along the lateral and polar surfaces of the bacteria. When co-cultured with AGS cells, neither the Δ*cagL* nor the Δ*cagI* mutant strains produced detectable pili (Figure 6C, F). This defect was rescued by complementation of the Δ*cagI* and Δ*cagL* mutants (Figure 6D, E, G).

**Table 3:** Analysis of type IV secretion system pili.

	Width (nm) a	Length (nm) a	Pili/cell^a^
**WT**	13.7±2.4	65.1±30.7	3.8±2.6
**Δ** ***cagH***	21.5±4.3 ***	115.1±65.1 ***	8.0±4.3 ***
**Δ** ***cagH*** **/CagH-HA**	14.4±2.8	65.6±29.9	3.3±2.9
**Δ** ***cagH*** **/CagH-HAΔCT**	15.8±5.1	53.4±18.5	3.5±2.7
**Δ** ***cagI*** **/CagI**	15.7±3.8	91.1±46.1	3.1±1.7
**Δ** ***cagL*** **/CagL-HA**	14.6±2.8	81.7±27.2	3.8±2.8

a Quantification of pilus width, length, and number is based on a minimum of 2 biological replicates and a minimum of 5 representative micrographs containing a total of at least 20 adherent *H. pylori* cells.

***, p<0.001 compared to WT, based on two-tailed T-test analysis.

**Figure 7:**
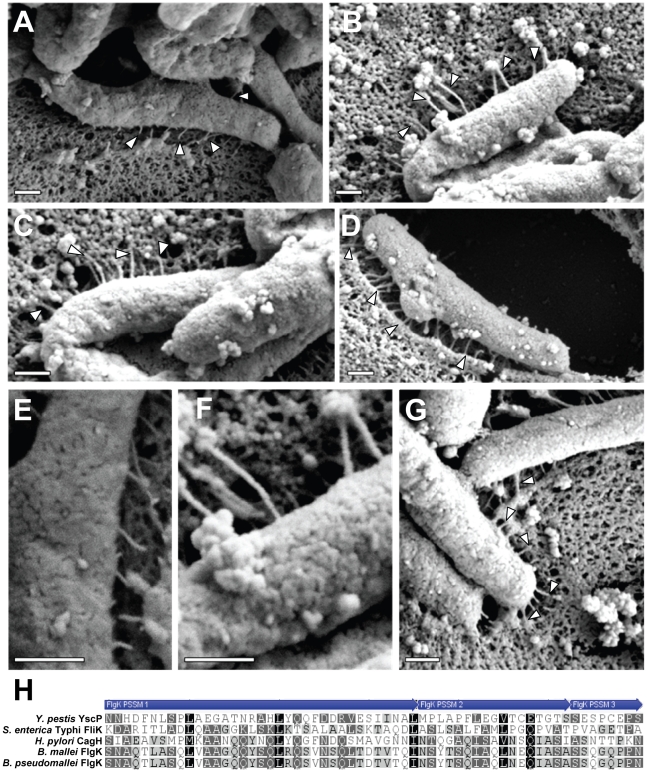
Analysis of pili formed by a Δ*cagH* mutant strain. WT *H. pylori*, a Δ*cagH* mutant, and a complemented mutant (Δ*cagH*/CagH-HA) were co-cultured with AGS cells and then analyzed by FESEM. **(A)** WT strain. **(B, C, D)** Multiple images of the Δ*cagH* mutant. **(E)** High magnification of panel A (WT strain). **(F)** High magnification of panel B (Δ*cagH* mutant). **(G)** Δ*cagH*/CagH-HA. Magnification bars indicate 200 nm. **(H)** Use of a position-specific scoring matrix (PSSM) within the NCBI Conserved Domain Database [57] identified a conserved flagellar hook protein K (FlgK) domain in CagH. The alignment compares the region of CagH corresponding to the FlgK domain with sequences of *Burkholderia* spp. FlgK and sequences of two members of the flagellar hook superfamily that function as molecular rulers (FliK from *Salmonella* and YscP from *Yersinia pestis*) [59], [60], [61], [64]. Arrows above the alignment indicate three portions of the FlgK domain identified in CagH.

In contrast to the Δ*cagI* and Δ*cagL* mutant strains, the Δ*cagH* mutant strain was capable of producing pili. However, there were several striking differences when comparing the Δ*cagH* mutant strain (Figure 7B, C, D, F) with the WT strain (Figure 7A, E). First, the number of pili produced by the Δ*cagH* mutant was significantly higher than the number of pili produced by the WT strain (8.0±4.3 vs. 3.8±2.6 visible pili per adherent bacteria, p<0.0001) (Table 3). Second, the length of pili produced by the Δ*cagH* mutant was significantly greater than that of pili produced by the WT strain (115.1±65.1 nm vs. 65.1±30.7 nm, p<0.0001). Third, the width of pili produced by the Δ*cagH* mutant was significantly greater than that of pili produced by the WT strain (21.5±4.3 nm vs. 13.7±2.4 nm, p<0.0001). A complemented Δ*cagH* mutant strain produced pili that were indistinguishable from the pili produced by the WT strain (Figure 7G and Table 3). These results suggest that CagH is a regulator of pilus dimensions. As considered further in the Discussion, an analysis of highly conserved domains within CagH (Figure 7H) provides clues into possible mechanisms by which CagH controls dimensions of the *H. pylori* T4SS pilus.

In all of the initial experiments (Tables 1, 2, and S2), interactions among CagH, CagI, and CagL were detected in *H. pylori* that were cultured in the absence of gastric epithelial cells. Our electron microscopy experiments indicate that pili associated with the *cag* T4SS are not detected when the bacteria are cultured in the absence of gastric epithelial cells (data not shown), and in agreement with previous reports [38], [39], we found that contact of *H. pylori* with gastric epithelial cells stimulates the formation of pili that are associated with the *cag* T4SS. This suggests that the *cag* T4SS is not yet fully assembled when bacteria are cultured in the absence of gastric epithelial cells. To determine whether interactions among CagH, CagI, and CagL are detectable under conditions in which the *cag* T4SS is fully assembled, we sought to detect the presence of this subassembly in *H. pylori* that were co-cultured with gastric epithelial cells. We co-cultured bacteria expressing CagH-HA with AGS cells, affinity purified CagH-HA from adherent bacteria, and then analyzed the protein content of the affinity purified preparation. Wild-type bacteria were co-cultured with AGS cells and were processed in parallel as a control. As shown in Table S4, we again observed co-purification of CagH, CagI, and CagL. Therefore, a CagH-CagI-CagL subassembly is detectable not only when bacteria are cultured in the absence of gastric epithelial cells, but also under conditions in which the *cag* T4SS is fully assembled.

Since CagL was previously detected as a pilus component [39], we hypothesized that CagI and CagH might also localize to pili. To test this hypothesis, we used scanning EM and immunogold labeling studies to analyze *H. pylori* that were co-cultured with gastric epithelial cells. Using this approach, we were unable to detect localization of CagH or CagI to the pili, and we were also unable to detect CagH or CagI in any other sites. Since we were able to detect surface localization of CagH, CagI, and CagL in transmission EM experiments and flow cytometry experiments but not FESEM experiments, there are probably limitations associated with the use of these monoclonal antibodies for immunogold labeling in the context of FESEM. Specifically, the FESEM methodology requires multiple extra washing steps (a series of seven sequential ethanol dehydration steps and three liquid carbon dioxide washing steps) that are not required for transmission EM, and monoclonal antibodies often are considered suboptimal compared to polyclonal antisera for immunogold EM studies [53].

### Analysis of a Conserved C-terminal Motif

**Figure 8:**
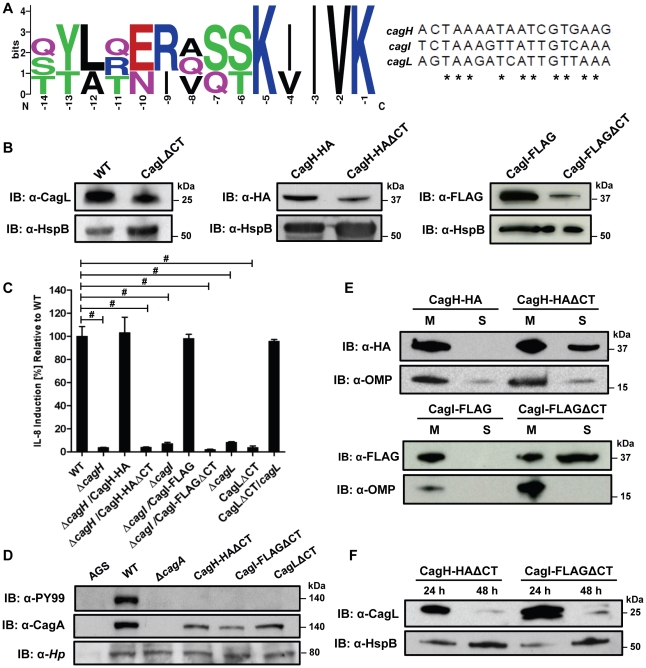
Analysis of a conserved C-terminal motif in CagH, CagI, and CagL. **(A)** A conserved hexapeptide C-terminal motif is present in CagH, CagI, and CagL (WebLogo [84]). Numbers indicate positions of amino acid residues relative to the C-terminus of CagH, CagI, or CagL. This C-terminal motif is encoded by divergent DNA sequences in *cagH, cagI*, and *cagL*. **(B)** Immunoblot analysis of mutants expressing C-terminally truncated forms of CagL, CagH-HA, or CagI-FLAG, each lacking a six-residue C-terminal motif. CagLΔCT was expressed from the endogenous *cagL* locus in the *cag* PAI, and CagH-HAΔCT and CagI-FLAGΔCT were expressed from the *ureA* locus. CagL expression was detected with anti-CagL serum, CagH-HA expression was detected with an anti-HA monoclonal antibody, and CagI-FLAG expression was detected with an anti-FLAG antibody. Truncated forms of CagL, CagH, and CagI were detected in each of the mutant strains, but the levels of the truncated proteins were reduced compared to levels of the full-length proteins. **(C)** AGS cells were co-cultured with WT *H. pylori* or the indicated mutants, and IL-8 expression was analyzed by ELISA as described in Methods. Values from six replicate samples were compared to the wild-type control by ANOVA followed by Dunnett's *post hoc* correction; # indicates p<0.05. **(D)** AGS cells were co-cultured with WT *H. pylori* or the indicated mutants, and tyrosine-phosphorylated CagA was detected with an anti-phosphotyrosine antibody (α-PY99). **(E)** Bacteria expressing CagH-HA, a C-terminally truncated form of CagH-HA (CagH-HAΔCT), CagI-FLAG, or a C-terminally truncated form of CagI (CagI-FLAGΔCT) were fractionated into total membrane (M) and soluble (S) fractions as described in Methods, and the tagged proteins were detected by immunoblotting. Intact CagH-HA and CagI-FLAG proteins were detected mainly in the membrane fraction. Deletion of the C-terminal motif from CagH-HA (CagH-HAΔCT) or CagI (CagI-FLAGΔCT) results in partial mislocalization of these proteins to the soluble fractions. **(F)** Bacteria expressing C-terminally truncated CagH (CagH-HAΔCT) or CagI (CagI-FLAGΔCT) were cultured for 24 or 48 h prior to lysis and immunoblotting to detect CagL. HspB was monitored as a loading control. At the 48 h timepoint, CagL levels are markedly reduced in strains expressing truncated CagH or CagI, compared to strains expressing full-length proteins.

Careful inspection of the sequences of CagH, CagI, and CagL revealed that all three proteins contain a conserved C-terminal motif (Figure 8A) consisting of the distal six amino acids of each protein. The C-terminal motifs of CagH, CagI, and CagL are encoded by divergent DNA sequences (Figure 8A). To investigate whether this C-terminal motif is functionally important, we generated *H. pylori* mutant strains expressing forms of CagH, CagI, or CagL in which this hexapeptide motif was deleted, as described in Methods. Deletion of the C-terminal motif individually in CagH, CagI, and CagL resulted in reduced levels of the mutated proteins compared to levels of the corresponding WT proteins (Figure 8B), along with a marked reduction in ability of the mutant bacteria to induce IL-8 secretion by AGS cells (Figure 8C) and abolishment of CagA translocation (Figure 8D). In contrast to wild-type CagH-HA and CagI-FLAG, which each localize to the membrane fraction (Figures 4 and 8E), CagH-HAΔCT and CagI-FLAGΔCT are partially mislocalized to the soluble fraction (Figure 8E). Furthermore, in the absence of the C-terminal motif of CagH-HA or CagI-FLAG, CagL stability is markedly reduced at the 48 h growth timepoint compared to CagL stability at the 24 h growth timepoint (Figure 8F). This result further supports the conclusion that CagH, CagI, and CagL are all members of a protein subassembly.

**Figure 9:**
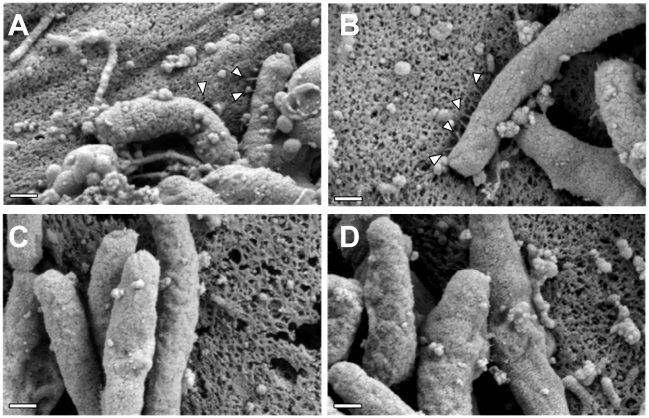
The C-terminal motif of CagI and CagL is required for pilus formation. WT *H. pylori* or mutant strains expressing C-terminally (CT) truncated forms of CagH, CagI, or CagL were co-cultured with AGS cells and imaged by FESEM. **(A)** WT. **(B)** CagH-HAΔCT. **(C)** CagI-FLAGΔCT. **(D)** CagLΔCT. Magnification bars indicate 200 nm.

Finally, we conducted experiments to determine whether the C-terminal motifs found in CagH, CagI, and CagL were required for formation of pili at the interface between *H. pylori* and gastric epithelial cells. Mutant strains lacking the C-terminal motif in CagH, CagI, or CagL were co-cultured with AGS cells, and the samples were then analyzed by FESEM. Bacteria expressing CagI-FLAGΔCT or CagLΔCT each failed to produce detectable pili (Figure 9C, D), and thus had a phenotype indistinguishable from Δ*cagI* and Δ*cagL* mutants (Figure 6). Bacteria expressing CagH-HAΔCT expressed pili that were indistinguishable from those produced by the WT strain (Figure 9A, B and Table 3). Despite producing normal-appearing pili, the CagH-HAΔCT mutation rendered the *cag* T4SS non-functional (Figure 8C, D). These experiments indicate that physical contact of these *H. pylori* pili with the AGS cell surface is not sufficient to induce IL-8 production, and indicate that the C-terminal motif present in CagH, CagI, and CagL is critical for functionality of the T4SS.

## Discussion

One of the important mechanisms by which *H. pylori* infection leads to severe gastric disease is through the actions of the bacterial oncoprotein CagA [4], [10], [11], [54]. Translocation of CagA into gastric epithelial cells occurs through a T4SS-mediated process and requires multiple proteins encoded by the *cag* PAI [12], [14]–[17], [21]. Several *H. pylori* proteins required for CagA translocation are distantly related to components of T4SSs in other bacterial species and presumably have conserved functions [12], [21], [28], [43]. In the current study, we provide new insights into three components of the *cag* T4SS that lack homologs in other T4SSs – CagH, CagI, and CagL.

Prior to the current study, it was known that CagL can bind α5β1 integrin and can cause several alterations in host cells [21], [39], [42], [44], [45]. CagL was localized in various studies to several bacterial subcellular sites, including a soluble bacterial fraction [38], [43], the bacterial surface [42], and pili on the surface of *H. pylori* [39] We reasoned that CagL might physically interact with other T4SS components, and that such interactions might be required for CagL export, localization, stability, or activity. Therefore, we conducted studies designed to identify *H. pylori* proteins that interact with CagL. In initial experiments involving affinity purification of CagL, we observed a highly reproducible co-purification of CagH and CagI with CagL, and we subsequently detected the same pattern of co-purification when either CagH or CagI was targeted for purification. We detected evidence of CagH-CagI-CagL interactions not only in experiments involving bacteria grown in pure culture, but also in experiments involving *H. pylori* that were attached to gastric epithelial cells. We were unable to undertake analysis of direct interactions among CagL, CagI and CagH because recombinant CagI and CagH could not be expressed in a soluble form under non-denaturing conditions. However, several other results provided evidence of a physical relationship among these proteins. Specifically, CagL stability was decreased in the absence of either CagI or CagH, and co-purification of CagI or CagH with CagL was dependent on the presence of all three proteins. All three proteins are required for functional activity of the *cag* T4SS, and all three proteins have a role in formation of pili at the interface between *H. pylori* and gastric epithelial cells. Other intriguing relationships among these proteins include their linkage within a single operon in the *cag* PAI [46], ability of both CagI and CagL to bind β1 integrin [39], [44], and presence of a nearly identical C-terminal motif in all three proteins. Collectively, the data suggest that CagL, CagI, and CagH are components of one or more T4SS subassemblies involved in pilus biogenesis.

Recognition of the relationships among CagH, CagI, and CagL in the current study was facilitated by using immunoaffinity purifications and a robust multidimensional mass spectrometry protein identification technique. In comparison to traditional co-immunoprecipitation experiments, which rely upon the availability of antibodies to detect potential interacting partners of a target protein by immunoblotting, the mass spectrometry-based approach used in the current study allowed a comprehensive analysis that was not limited by the availability of antibodies. In addition, this approach affords advantages compared to two-hybrid screens, because it allows detection of interactions among native proteins expressed in *H. pylori,* as well as the potential to detect indirect secondary and tertiary interactions. Analysis of the appropriate control strains allowed for assessment of non-specific protein interactions and statistical evaluation of results.

Prior to the current study, CagH and CagI had not been investigated in detail. Neither CagH nor CagI was detected in analyses of *H. pylori* using 2D gel proteomic methodology [35], [55], and immunologic detection of CagI expression has been hindered by difficulty in raising antibodies against the protein [44]. Similarly, our efforts to raise polyclonal antisera against CagH or CagI proteins failed to yield antisera that recognized the appropriate antigens. However, we were able to detect CagH and CagI expression by mass spectrometry, and we were also able to detect epitope-tagged forms of these *H. pylori* proteins. One study reported that CagI was capable of binding β1 integrin in a yeast two hybrid assay, and CagI interactions with integrin were confirmed by analyzing binding of a CagI-GST fusion protein to cell lines deficient in β1 integrin expression or cell lines that had been genetically complemented with human β1 integrin [44]. Another study reported that *H. pylori* mutant strains containing transposon insertions in *cagH* or *cagI* were defective in their ability to translocate CagA into host cells, and that CagH (but not CagI) was required for induction of IL-8 synthesis and secretion by cultured gastric epithelial cells [21]. In the current study, we found that *cagH* and *cagI* mutant strains were defective in both CagA translocation and IL-8 induction. The discrepancy in results pertaining to CagI and IL-8 induction might be attributable to differences in the mutagenesis methods utilized to generate Δ*cagI* isogenic mutants. In the current study, we analyzed unmarked strains containing deletions of the relevant *cag* genes, and complementation of the unmarked mutants confirmed that CagH and CagI are required for CagA translocation and stimulation of IL-8 secretion by cultured gastric epithelial cells.

Localization analyses performed in the current study indicated that CagH, CagI, and CagL can all be exported to the surface of *H. pylori,* and in addition, provided evidence for the existence of non-surface-exposed pools of these proteins. The existence of these proteins in multiple subcellular locations potentially reflects multiple stages of T4SS assembly. Detection of CagL on the surface of *H. pylori* in the current study agrees with previous reports, which reported localization of CagL to either the bacterial surface or to pilus structures, based on immunogold EM staining [39], [42]. Other lines of evidence supporting a surface-exposed localization of CagI and CagL include the ability of both proteins to bind β1 integrin [39], [44], signatures of positive selection in the genes encoding these two proteins [56], and the presence of a predicted signal sequence in both proteins (Figure 1E). The detection of a non-surface-exposed pool of CagL in the current study agrees with results of previous studies, which detected CagL in a soluble bacterial fraction consistent with the periplasm [38], [43].

Determining the subcellular location where CagH, CagI, and CagL assemble into complexes is complicated by the presence of these proteins in multiple subcellular sites. All three proteins can localize to the bacterial surface, and this is one site where the formation of CagH-CagI-CagL complexes may be relevant. In addition, since a large proportion of CagL is localized to a soluble fraction, it seems likely that periplasmic CagL may interact with membrane-associated CagH or CagI proteins that have domains in the periplasm. We detected evidence of CagH-CagI-CagL complexes not only in pure bacterial cultures, but also in co-cultures of *H. pylori* with gastric epithelial cells (Tables 1, 2, S4). Since the CagH-CagI-CagL complexes were detected in pure bacterial cultures (which lack detectable surface pili), the complexes must clearly be localized to sites distinct from pili under these conditions. A previous study used immunogold labeling methods and a polyclonal antiserum to detect localization of CagL to pilus structures [39]. In the current study, we were not able to detect localization of CagH or CagI to pilus structures, probably due to limitations associated with the use of monoclonal antibodies for immunogold labeling in the context of scanning electron microscopy. Specifically, the FESEM methodology requires multiple extra washing steps (a series of seven sequential ethanol dehydration steps and three liquid carbon dioxide washing steps) that are not required for transmission EM, and monoclonal antibodies often are considered suboptimal compared to polyclonal antisera for immunogold EM studies [53]. Therefore, at present it is not known whether CagL localization to pili requires dissociation of CagL from CagH and CagI, or whether all three proteins eventually localize to pili.

An important finding in the current study is the demonstration that both CagL and CagI are required for formation of pili. Complementation of Δ*cagL* and Δ*cagI* mutant strains resulted in restoration of pilus formation and T4SS function. In previous studies, CagY, CagT, and VirB11 ATPase were reported to be required for formation of pili [38], [39], [41], but the role of these proteins in *H. pylori* pilus formation has not yet been verified by testing of complemented mutant strains. CagT, CagY, and the VirB11 ATPase are each homologous to proteins found in T4SSs of other bacteria, but in contrast, CagI and CagL do not have homologues in other T4SSs.

Interestingly, a Δ*cagH* mutant strain was hyperpiliated and expressed pili that were elongated and thickened in comparison to pili of the WT strain. The increased number of pili visualized in images of the Δ*cagH* mutant strain might be attributable to their increased thickness and decreased fragility, resulting in a greater likelihood that the structures are preserved and visualized. Complementation of the Δ*cagH* mutant strain resulted in production of pili with a WT morphology. These results suggest that CagH is a regulator of pilus dimensions. Analysis of the CagH sequence using a conserved domain database search [57] (NCBI) indicates that it contains a flagellar hook-associated protein (FlgK) domain (PRK06945) (Figure 7H). The FlgK domain within CagH is most closely related to the corresponding domains found in FlgK proteins of *Burkholderia* spp., rather than the corresponding domain found in *H. pylori* FlgK. The FlgK domain in CagH also exhibits similarity to portions of the FliK flagellar protein of *Salmonella* and the YscP type III secretion system protein of *Yersinia pestis* (Figure 7H), two proteins that are known to be determinants of either flagellar hook length [58]-[63] or type III secretion system needle length [64]. We speculate that, analogous to the role of FlgK (a flagellar hook-junction protein) in terminating flagellar hook assembly, CagH may have a role in terminating pilus assembly. Alternatively, analogous to FliK and YscP, CagH may serve as a molecular ruler to control the dimensions of *cag* T4SS pili in *H. pylori*. It will be important in future studies to dissect the molecular mechanisms by which CagH regulates pilus dimensions.

Similar to previous studies [38]-[41], we observed that *H. pylori* contact with gastric epithelial cells stimulated the production of pili, and in agreement with previous studies [39], [41], we observed that Δ*cag* PAI mutant, *cagT* mutant, and *cagE* mutant strains failed to produce pili. However, the dimensions of the pili visualized in the current study differed from what was reported previously. Specifically, a previous study [38] reported that the pili were between 45 and 75 nm in width, depending on presence or absence of a sheath structure, whereas in the current study, we found that the pili were much thinner. The same strain of *H. pylori* was used in both studies, so this difference is probably not attributable to strain-dependent variation. We speculate that the difference in reported dimensions might be attributable to differences in methods for scanning EM, such as the use of a relatively thin layer of gold in the current study and a thicker layer of coating in the previous study.

A striking feature shared by CagH, CagI, and CagL is a conserved C-terminal hexapeptide motif (Figure 8A). This C-terminal motif is not found in any other protein encoded by *H. pylori* strain 26695. In the current study, we show that this motif is required for functional activity of the T4SS, and that the C-terminal motifs of CagI and CagL are required for pilus formation. Deletion of the C-terminal motif leads to partial mislocalization of CagH and CagI to a soluble fraction instead of a membrane fraction (Figure 8E). Based on the observed mislocalization of C-terminally truncated CagH and CagI proteins, we hypothesize that this C-terminal motif plays a role in protein sorting or localization. In several other bacterial species, C-terminal motifs are required for recognition of proteins by the T4SS machinery. For example, RalF, an effector molecule of the *Legionella* Dot/Icm system, has a C-terminal motif consisting of hydrophobic residues flanked by lysine/arginine moieties [65]. Mutagenesis studies indicated that deletion of the distal 3 terminal amino acid residues of RalF leads to loss of recognition by the T4SS machinery, and thus loss of translocation [65]. C-terminal translocation signals in T4SS substrates also have been detected in *A. tumefaciens* and *Bartonella henselae* [66], [67]. In a similar manner, we propose that the conserved C-terminal motifs in CagH, CagI, and CagL target these proteins to appropriate sites within the *H. pylori* T4SS. Interestingly, a mutant strain expressing a C-terminally truncated form of CagH produced normal-appearing pili, but this strain was nevertheless defective in both CagA translocation and ability to stimulate IL-8 induction. This result reveals that contact of these *H. pylori* pili with the AGS cell surface is not sufficient to induce IL-8 production.

T4SSs are a diverse collection of macromolecular machines, found in a broad phylogenetic range of bacteria. It has been proposed that all T4SSs employ a common mechanism for secretion across the cytoplasmic membrane, and that diversity has arisen primarily as a consequence of variation in the types of cell envelopes that must be spanned, along with variation in donor-host cell interactions [29]. Our current understanding of the architecture of T4SSs is based on elegant work with several model systems, including the VirB/VirD4 system of *A. tumefaciens* and several plasmid conjugation systems. There are limitations, however, when comparing these model systems with the T4SSs found in distantly related bacteria. Several components of the *H. pylori cag* T4SS are distantly related to components of the VirB/VirD4 system, and as highlighted in the current study, other key constituents of the *cag* T4SS lack homology to components of T4SSs in other bacterial species. In addition to the three *H. pylori* proteins analyzed in the current study, *cag* T4SS functionality probably requires at least six other proteins that lack homology to VirB/VirD4 proteins [21]. In future studies, it will be important to investigate the functional roles of these proteins in further detail, and from a broader perspective, it will be important to investigate the structural correlates of biological diversity among T4SSs.

## Methods

### Bacterial Strains and Growth Conditions

*H. pylori* 26695 and its *cag* isogenic mutant derivative strains were grown on trypticase soy agar plates supplemented with 5% sheep blood or Brucella agar plates supplemented with 5% fetal bovine serum at 37°C in room air containing 5% CO_2_. *H. pylori* mutant strains were selected based on resistance to chloramphenicol (5 µg/ml), kanamycin (10 µg/ml), or metronidazole (7.5 to 15 µg/ml). *E. coli* strain DH5α, used for plasmid propagation, was grown on Luria-Bertani agar plates or in Luria-Bertani liquid medium supplemented with ampicillin (50 µg/ml), chloramphenicol (25 µg/ml), or kanamycin (25 µg/ml), as appropriate.

### Generation of Rabbit Polyclonal anti-CagL Serum

CagL derived from *H. pylori* 26695 (and lacking a putative signal sequence) was expressed as a GST fusion protein from pGEX-6P-1 vector (GE Healthcare, formerly Amersham). CagL-GST was purified using gluthathione beads [35]. Rabbits were then immunized with purified CagL-GST.

### Immunoblot Analysis

To detect expression of Cag proteins, individual samples were separated by SDS-PAGE (4-20% gradient), transferred to a nitrocellulose membrane, and subsequently immunoblotted using rabbit polyclonal antiserum raised against recombinant CagL, or mouse monoclonal antibodies reactive with FLAG or HA epitopes (Sigma). To confirm similar loading of samples, immunoblotting using either a monoclonal mouse antibody (Santa Cruz) or a rabbit polyclonal antiserum to *H. pylori* HspB, a GroEL homolog, was utilized [68]. Horseradish peroxidase-conjugated anti-rabbit IgG or anti-mouse IgG was used as the second antibody. Signals were generated by enhanced chemiluminescence reaction and detection by exposure to X-ray film.

### Cell Culture Methods

AGS human gastric epithelial cells were grown in the presence of 5% CO_2_ in RPMI medium containing 10% FBS, 2 mM L-glutamine, and 10 mM HEPES buffer.

### IL-8 Secretion by Gastric Cells in Contact with *H. pylori*

*H. pylori* strains were co-cultured with AGS cells at a multiplicity of infection of 100:1, and IL-8 secretion was analyzed using an anti-human IL-8 sandwich ELISA (R&D). Levels of IL-8 secreted by AGS cells in contact with isogenic *cag* mutants were compared to levels secreted by AGS cells in response to infection with WT *H. pylori* 26695.

### CagA Translocation Assay

Translocation of CagA into AGS cells was analyzed by co-culturing *H. pylori* strains with AGS cells and detecting tyrosine phosphorylation of CagA, as previously described [35], [69]. Briefly, *H. pylori* and AGS human gastric cells were co-cultured at a MOI of 100:1 for 24 h at 37°C. Cells were lysed in NP-40 lysis buffer containing Complete Mini EDTA-free Protease Inhibitor (Roche) and 2 mM sodium orthovanadate, and CagA translocation was assessed by separating the soluble fraction using 7.5% SDS-PAGE and immunoblotting with an anti-phosphotyrosine antibody (α-PY99, Santa Cruz).

### Synthesis of a *cat-rdxA* Cassette

To facilitate introduction of unmarked mutations into *H. pylori* chromosomal genes of interest, a *cat-rdxA* cassette was synthesized and cloned into pUC57 vector (Genscript). This cassette confers resistance to chloramphenicol mediated by the chloramphenicol acetyl-transferase (*cat*) gene from *Campylobacter coli*, and susceptibility to metronidazole is mediated by an intact *rdxA* gene (HP0954) from *H. pylori* 26695. Both genes are in the same orientation, with *cat* upstream from *rdxA*. Expression of *cat* is driven by the *C. coli cat* promoter, and expression of *rdxA* is driven by the *H. pylori vacA* promoter. The two genes are separated by a presumed *C. coli cat* terminator, and a ФT7 terminator is located at the 3′-distal end of the *rdxA* gene.

### Mutagenesis of *H. pylori cag* Genes

To construct a Δ*cagL* mutant strain, we PCR-amplified *cagL* along with approximately 0.5 kb of flanking DNA from *H. pylori* 26695 genomic DNA using Amplitaq Gold (ABI), and cloned the PCR product into pGEM-T Easy (Promega). By using this plasmid as a template for inverse PCR and then ligating the PCR product, we generated a modified plasmid lacking *cagL* and containing a BamHI site at the site of the deletion. A kanamycin resistance cassette was cloned into the BamHI site to yield pCSLK2-4. *H. pylori* 26695 was transformed with pCSLK2-4 (which is unable to replicate in *H. pylori*), and single colonies resistant to kanamycin were selected. PCR and sequencing were used to confirm that the kanamycin cassette had inserted into the *cagL* locus in the same orientation as operon transcription.

To generate unmarked mutant strains, we used a new method that is a variant of counterselection methods used previously in *H. pylori* (Figure 2) [70], [71]. As a first step, the *rdxA* gene, which confers resistance to metronidazole, as well as approximately 0.5 kb of flanking DNA on each side, was PCR-amplified from *H. pylori* 26695 and cloned into pGEM-T Easy to yield pMM670. By using pMM670 as the template for inverse PCR and then ligating the PCR product, we generated a modified plasmid (pMM672) in which the coding region of *rdx*A was deleted. Transformation of *H. pylori* 26695 with pMM672 (which is unable to replicate in *H. pylori*) and selection for metronidazole-resistant colonies resulted in the recovery of a mutant in which the *rdx*A locus was deleted (*H. pylori* Δ*rdxA*) (Figure 2A).

For each *cag* gene of interest, we PCR-amplified the relevant gene as well as approximately 0.5 kb of flanking DNA on each side from *H. pylori* 26695 genomic DNA, and cloned these sequences into pGEM-T Easy. By using these plasmids as templates for inverse PCR and then ligating the PCR products, we generated modified plasmids lacking the *cag* gene of interest and containing a BamHI site at the site of the deletion. A *cat-rdxA* cassette (described above) was then cloned into the BamHI site. Plasmids containing the *cat-rdxA* cassette (which are unable to replicate in *H. pylori*) were transformed into *H. pylori* Δ*rdxA*, and single colonies resistant to chloramphenicol and susceptible to metronidazole were selected (Figure 2A, B). In each case, PCR analysis confirmed that the *cat-rdxA* cassette had inserted into the desired chromosomal site and that the relevant *cag* gene was deleted. To generate unmarked mutants, strains containing the *cat-rdxA* cassette were transformed with plasmids harboring the desired mutations, and transformants resistant to metronidazole were selected (Figure 2A, B). A similar approach was used to introduce a gene encoding a C-terminally truncated form of CagL into the endogenous *cagL* locus.

To complement mutants in *cis* at a heterologous chromosomal locus, we used a plasmid derived from pAD1 [72], which allows genes of interest to be introduced into the *H. pylori* chromosomal *ureA* locus. The modified pAD1 plasmid contains a chloramphenicol resistance cassette, restriction sites to allow cloning of a gene of interest into a site downstream from the *ureA* promoter and a ribosomal binding site, and flanking sequences derived from the *ureA* locus. Plasmids were constructed to allow expression of epitope-tagged forms of CagH, CagI, and CagL, and in addition, plasmids were constructed to allow expression of untagged forms of CagI and CagL. CagL was expressed as a protein containing a hemagglutinin (HA) tag introduced at residue 22 immediately following a putative signal sequence (Figure 1D). CagH was expressed as a protein containing an N-terminal HA tag, and CagI was expressed with an internal FLAG epitope introduced at residue 51 in a region downstream of the predicted signal sequence (Figure 2C), which is predicted to be surface exposed based on hydrophilicity plots (ProtScale in Expasy) [73]. *H. pylori* strains were transformed with these plasmids, and chloramphenicol-resistant colonies were selected. Expression of the epitope-tagged proteins was verified by immunoblotting using monoclonal anti-HA or anti-FLAG antibodies, respectively.

### Immunoaffinity Purification of Cag Proteins

WT or Δ*cagL* isogenic mutant bacteria cultured on solid media were harvested after 48 h of growth, washed once in phosphate-buffered saline (PBS), and lysed overnight at 4°C in RIPA buffer (10 mM Tris, 100 mM NaCL, 1% NP-40, 0.25% deoxycholic acid, Complete Mini EDTA-free Protease Inhibitor (Roche), pH 7.2). Cellular debris was removed by high speed centrifugation. CagL was immunoaffinity purified from bacterial lysate using polyclonal anti-CagL antibodies that had been covalently cross-linked to a Protein A support (Dynal, Invitrogen). Alternatively, CagL-HA and CagH-HA were purified from strains expressing these proteins using monoclonal anti-HA antibodies (Sigma) that had been either covalently cross-linked or non-covalently bound to a Protein G support (Dynal, Invitrogen). Immobilized anti-FLAG M2 antibody (Sigma) was utilized for immunoaffinity purification of CagI-FLAG. Target proteins were immunoaffinity purified at room temperature. Immunoaffinity purified proteins were washed in 100 bed volumes of PBS containing 0.1% Tween-20 (PBST 0.1%) prior to elution by boiling in SDS buffer (0.3 M Tris-HCl, 1% SDS, 10% glycerol, 100 mM DTT, Pierce), or elution by HA or FLAG peptide competition (1 mg/ml in PBS for HA peptide [Sigma], 1 mg/ml in TBS for FLAG peptide [Sigma]). In each case, the presence of the targeted protein in the immunoaffinity purified sample was verified by immunoblotting.

To purify Cag proteins from bacteria attached to gastric epithelial cells, the WT strain and a strain expressing CagH-HA were each co-cultured with AGS cells (80% confluent) at an MOI of 100 for 5 hours prior to the addition of RIPA buffer and mechanical disruption of the cell monolayers. Cells were lysed by 3 pulses of sonication (10 s for each pulse), followed by overnight incubation at 4°C in RIPA buffer. Cleared lysates were incubated with immobilized anti-HA monoclonal antibody (Sigma) at room temperature. Immunoaffinity-purified preparations were washed with 100 bed volumes of PBST 0.1%. Bound proteins were eluted by HA peptide competition.

### Mass Spectrometric Analysis of Samples

To provide a comprehensive analysis of the protein content in immunoaffinity purified samples, the samples were analyzed by multidimensional protein identification technology (MudPIT). Purified proteins were eluted from the beads, run about 2 cm into a 10% NuPAGE gel, and then subjected to in-gel digestion with trypsin and peptide extraction. The resulting peptide mixtures were analyzed via MudPIT essentially as described [74]. Briefly, peptides were loaded via pressure cell (New Objective) onto a biphasic pre-column fritted using an Upchurch M-520 filter union (IDEX). This 100-µm fused silica microcapillary column was packed with 3 cm of 5-µm C18 reverse-phase resin (Jupiter, Phenomenex) followed by 5 cm of strong cation-exchange resin (Luna SCX, Phenomenex). Once loaded, it was then placed in-line with a 100 µm×20 cm, C18 packed emitter tip column (Jupiter C18, 3 µm, 300 Å, Phenomonex) coupled to an LTQ ion trap mass spectrometer equipped with an Eksigent NanoLC-AS1 Autosampler 2.08, an Eksigent NanoLC-1D plus HPLC pump, and nanospray source. separations were accomplished using 5 µl autosampled pulses of ammonium acetate in 0.1% formic acid (25, 50*, 75, 100, 150*, 200, 250*, 300, 500*, 750*, 1000* mM pulses - * shorter MudPITs containing these salt pulses were performed on isolated proteins) followed by a 105 min reversed phase gradient from water 0.1% formic acid to 45% acetonitrile 0.1% formic acid. Tandem mass spectra were collected throughout the runs in a data dependent manner using dynamic exclusion to improve data acquisition of lower intensity peptides. These spectra were extracted from the instrument files using ScanSifter and searched using SEQUEST [75] against an *H. pylori* strain 26695 database that also contained common contaminants and reversed versions of the *H. pylori* proteins. Identifications were filtered to an estimated desired false discovery rate (FDR) using reverse database hits and collated to proteins using IDPicker [76]. All reported proteins were identified with a minimum of 2 distinct peptides.

### Real-time PCR

Total RNA was isolated from *H. pylori* using Trizol Reagent (Gibco), according to the manufacturer's protocol. RNA samples were refined using the RNeasy Mini Kit (Qiagen), and on-column RNase-free DNase digestion. cDNA synthesis was performed on 100 ng of purified RNA using the iScript cDNA synthesis kit (BioRad). As a control, first strand cDNA reactions were carried out in parallel without reverse transcriptase. Real time PCR was executed in triplicate on an ABI StepOne Real Time PCR machine, using TaqMan MGB chemistry. Abundance of *cagH*, *cagI*, and *cagL* transcripts in *cag* isogenic mutant strains was calculated using the ΔΔCT method, with each transcript signal normalized to the abundance of the *recA* internal control and comparison to the normalized transcript levels of WT *H. pylori*.

### Bacterial Subcellular Fractionation

Wild-type *H. pylori* strain 26695, as well as strains expressing CagL-HA, CagH-HA or CagI-FLAG, were grown on solid media for 24 h prior to harvest, washing, and resuspension in sonication buffer (10 mM Tris-HCL, pH 8.0, Complete Mini EDTA-Free Protease Inhibitor (Roche)). Bacteria were lysed by 5 pulses of sonication. Unbroken bacteria and cellular debris were removed from the lysate by centrifugation at 4500 x g for 10 min. The supernatant was separated by ultracentrifugation (1 h at 250,000 x g, 4°C) into a soluble fraction and a total membrane fraction. Proteins in the soluble fraction were concentrated by methanol-chloroform precipitation [77] prior to solubilization in reducing SDS-PAGE buffer (Pierce). The total membrane fraction was washed three times in sonication buffer, and resuspended in reducing SDS-PAGE buffer (Pierce). Subcellular fractions were immunoblotted with anti-CagL, anti-HA and anti-FLAG antibodies, followed by appropriate secondary antibodies, to detect untagged CagL, CagL-HA, CagH-HA, and CagI-FLAG, respectively.

### Cleavage of Surface Proteins by Proteinase K

Susceptibility of surface-exposed *H. pylori* proteins to digestion with proteinase K was assessed using a modification of previous protocols [68]. *H. pylori* expressing CagH-HA, CagI-FLAG, or CagL-HA were grown for 24 h on blood agar plates prior to harvesting and washing in PBS. Bacteria were resuspended in RMPI medium, or RPMI medium containing proteinase K (0-20 µg/ml). Bacteria were incubated for 30 min on ice, and proteinase K activity then was abrogated by addition of PMSF (final concentration 2 mM). After washing in RPMI containing 2 mM PMSF, the bacteria were resuspended in SDS sample buffer and analyzed by SDS-PAGE and immunoblotting. CagH-HA, CagI-FLAG, and CagL-HA were detected with monoclonal anti-HA or monoclonal anti-FLAG antibodies. Cleavage of VacA, which is known to be present on the *H. pylori* surface [50], [51], was assessed using an anti-VacA antiserum [78]. Cleavage of carbonic anhydrase was assessed using an antiserum raised against a periplasmic domain of this protein [52]. Densitometry was accomplished using Image J software [79]; band densities were normalized to HspB and compared to control samples that were not treated with proteinase K.

### Flow Cytometry

Bacteria were grown overnight in Brucella broth supplemented with 5% fetal bovine serum. Bacterial cells were fixed in 4% paraformaldehyde before staining with primary antibody (monoclonal anti-HA [Sigma] or monoclonal anti-FLAG [Sigma]) in PBS supplemented with 50 mM EDTA and 0.1% BSA, followed by staining with secondary antibody (goat F(ab')_2_ anti-mouse IgG labeled with Alexa Fluor 488) in PBS supplemented with 0.1% BSA. Flow cytometry data were collected on a Becton Dickenson LSR2 instrument using Becton Dickenson FACS Diva 6.1.3 software. 20,000 single cell events were collected for each sample and analyzed using Tree Star Flow Jo 8.8.2 software.

### Immunoelectron Microscopy

*H. pylori* cells were grown on blood agar plates for 48 h as described above. Immunogold labeling was performed on whole bacterial cells as previously described with some modifications [41]. Briefly, bacterial cells were lifted onto formvar-coated 200 mesh copper TEM grids (Electron Microscopy Sciences). Cells were washed three times with pre-warmed PBS and incubated in 1% gelatin in PBS for 30 min at 37°C. After blocking, mouse monoclonal primary antibodies (anti-HA or anti-FLAG) were applied to the sample grids and incubated for 30 min at 37°C. Subsequently, grids were washed three times with pre-warmed PBS and a second blocking step was performed using 1% gelatin in PBS for 30 min at 37°C. Then goat anti-mouse secondary antibodies conjugated to 10 nm colloidal gold particles (Ted Pella, Inc.) were applied and incubated for 30 min at 37°C. Afterward, grids were washed three times with prewarmed PBS and cells were negatively stained with 1% ammonium molybdate before being visualized with a Philips CM-12 transmission electron microscope.

### FESEM of *H. pylori* in Contact with Gastric Epithelial Cells

*H. pylori* and AGS human gastric cells were co-cultured at a MOI of 100∶1 on tissue culture-treated coverslips (BD Biosciences) for 4 h at 37°C in the presence of 5% CO_2_. Cells were fixed with 2.0% paraformaldehyde, 2.5% glutaraldehyde in 0.05 M sodium cacodylate buffer for 1 h at 37°C. Coverslips were washed with sodium cacodylate buffer and secondary fixation was performed with 1% osmium tetroxide at room temperature for 2 h. Coverslips were washed with sodium cacodylate buffer and dehydrated with sequential washes of increasing concentrations of ethanol. Samples were then dried at the critical point, mounted onto sample stubs, grounded with a thin strip of silver paint at the sample edge, and sputter-coated with gold before viewing with a Zeiss Supra 35V FEG scanning electron microscope. Analysis of pilus dimensions and image analysis was performed using Image J software [79].

### Statistical Analysis

The statistical significance of differences in numbers of spectral counts of proteins detected in different immunoaffinity purified preparations was determined by G-test, as previously described [80]. The statistical significance of differences in IL-8 production or *cag* gene expression when comparing WT *H. pylori* with mutant strains was determined by one-way ANOVA followed by Dunnett's post-hoc test for multiple comparisons against a single control [81]. Statistical analysis of pilus dimensions was performed using a two-tailed Student's T-Test.

### Ethics Statement

This study was carried out in strict accordance with the recommendations in the Guide for the Care and Use of Laboratory Animals of the National Institutes of Health. The protocol was approved by the Institutional Animal Care and Use Committee of Vanderbilt University School of Medicine (M/07/292).

### Accession Numbers

*H. pylori* 26695 whole genome, GI:15644634; http://genolist.pasteur.fr/PyloriGene/; CagH, NP_207337; CagI, NP_207336; CagL, NP_207335.

## Supporting Information

Table S1 Cag proteins identified in *H. pylori* whole cell lysate.
(DOC)

Table S2 Proteins detected in immunoaffinity-purified preparations of CagH-HA and CagL-HA.
(DOC)

Table S3 Cag proteins that co-purify with CagL in the absence of CagH or CagI.
(DOC)

Table S4 Co-purification of CagH, CagI, and CagL from *H. pylori* attached to gastric epithelial cells.
(DOC)
