# Supplementary material for: Helicobacter pylori Exploits a Unique Repertoire of Type IV Secretion System Components for Pilus Assembly at the Bacteria-Host Cell Interface
Source: PLoS Pathog. 2011 Sep 1;7(9):e1002237. doi: 10.1371/journal.ppat.1002237 (PMC3164655; doi:10.1371/journal.ppat.1002237)
Supplement: Table S1 — Cag proteins identified in H. pylori whole cell lysate. (DOC) [file ppat.1002237.s001.doc]

**Table S1. Cag proteins identified in *H. pylori*** whole cell lysate

| Gene Number ^a^ | Protein | Homology ^b^ | SDS ^c^ | RIPA ^c^ |
| --- | --- | --- | --- | --- |
| HP0520 | Cag1 |  | 12 | 10 |
| HP0522 | Cag3 |  | 56 | 3 |
| HP0524 | Cag5 | VirD4 | 38 | 14 |
| HP0525 | Cagα | VirB11 | 16 | 4 |
| HP0526 | CagZ |  | 4 | 7 |
| HP0527 | CagY | VirB10 | 43 | 5 |
| HP0528 | CagX | VirB9 | 21 | 1 |
| HP0529 | CagW |  | 8 | 10 |
| HP0530 | CagV | VirB8 | 29 | 4 |
| HP0531 | CagU |  | 2 | 5 |
| HP0532 | CagT | VirB7 | 26 | 1 |
| HP0534 | CagS |  | 0 | 2 |
| HP0536 | CagP |  | 2 | 0 |
| HP0537 | CagM |  | 20 | 6 |
| HP0538 | CagN |  | 1 | 1 |
| HP0540 | CagI |  | 0 | 4 |
| HP0541 | CagH |  | 0 | 2 |
| HP0543 | CagF |  | 7 | 56 |
| HP0544 | CagE | VirB4 | 67 | 15 |
| HP0545 | CagD |  | 6 | 12 |
| HP0546 | CagC | VirB2 | 7 | 0 |
| HP0547 | CagA |  | 268 | 114 |
| Total Spectral Counts | |  | 27688 | 33569 |
| ^a^ Based on the *H. pylori* 26695 genome annotation | | |  |  |
| ^b^ Based on comparison to the *Agrobacterium tumefaciens* T4SS | | | |  |
| ^c^ *H. pylori* 26695 was cultured for 48 h and then solubilized in either 1% SDS or RIPA buffer. The Table shows numbers of raw spectral counts observed by MudPIT analysis for each identified Cag protein. | | | | |
