# Supplementary material for: Helicobacter pylori Exploits a Unique Repertoire of Type IV Secretion System Components for Pilus Assembly at the Bacteria-Host Cell Interface
Source: PLoS Pathog. 2011 Sep 1;7(9):e1002237. doi: 10.1371/journal.ppat.1002237 (PMC3164655; doi:10.1371/journal.ppat.1002237)
Supplement: Table S2 — Proteins detected in immunoaffinity-purified preparations of CagH-HA and CagL-HA. (DOC) [file ppat.1002237.s002.doc]

Table S2. Proteins detected in immunoaffinity-purified preparations of CagH-HA and CagL-HA

| **Gene Number ^a^** | **Protein** | **WT ^b^** | **CagH-HA ^b^** | | **CagL-HA ^b^** |
| --- | --- | --- | --- | --- | --- |
| HP0539 | CagL | 0 | 98 ^***^ | | 21 ^***^ |
| HP0540 | CagI | 0 | 203 ^***^ | | 10 ^***^ |
| HP0541 | CagH | 1 | 91 ^***^ | | 2 |
| HP0547 | CagA | 0 | 2 | | 0 |
| HP0535 | CagQ | 1 | 4 | | 0 |
| HP0010 | chaperone and heat shock protein | 16 | 72 | | 19 |
| HP0011 | co-chaperone | 0 | 4 | | 0 |
| HP0025 | outer membrane protein 2 | 1 | 9 | | 3 |
| HP0027 | isocitrate dehydrogenase | 1 | 4 | | 1 |
| HP0061 | hypothetical protein | 1 | 0 | | 1 |
| HP0072 | urease beta subunit | 7 | 0 | | 0 |
| HP0073 | urease, alpha subunit | 6 | 0 | | 0 |
| HP0082 | methyl-accepting chemotaxis transducer | 0 | 12 | | 0 |
| HP0083 | ribosomal protein S9 | 1 | 1 | | 0 |
| HP0086 | conserved hypothetical protein | 0 | 3 | | 0 |
| HP0099 | methyl-accepting chemotaxis protein | 1 | 22 | | 0 |
| HP0103 | methyl-accepting chemotaxis protein | 1 | 1 | | 0 |
| HP0109 | chaperone and heat shock protein 70 | 2 | 11 | | 1 |
| HP0111 | hypothetical protein | 1 | 1 | | 0 |
| HP0116 | DNA topoisomerase I | 0 | 7 | | 0 |
| HP0153 | recombinase | 0 | 8 | | 0 |
| HP0175 | cell binding factor 2 | 2 | 2 | | 1 |
| HP0176 | fructose-bisphosphate aldolase | 0 | 2 | | 0 |
| HP0197 | S-adenosylmethionine synthetase 2 | 0 | 5 | | 0 |
| HP0210 | chaperone and heat shock protein | 0 | 4 | | 0 |
| HP0224 | peptide methionine sulfoxide reductase | 2 | 5 | | 0 |
| HP0231 | hypothetical protein | 2 | 0 | | 1 |
| HP0243 | neutrophil activating protein | 12 | 0 | | 3 |
| HP0264 | ATP-dependent protease binding subunit | 0 | 8 | | 0 |
| HP0296 | ribosomal protein L21 | 1 | 7 | | 0 |
| HP0297 | ribosomal protein L27 | 1 | 1 | | 0 |
| HP0305 | hypothetical protein | 2 | 2 | | 0 |
| HP0310 | conserved hypothetical protein | 0 | 2 | | 0 |
| HP0317 | outer membrane protein 9 | 1 | 25 | | 1 |
| HP0319 | arginyl-tRNA synthetase | 1 | 1 | | 0 |
| HP0370 | biotin carboxylase | 0 | 3 | | 0 |
| HP0371 | biotin carboxyl carrier protein | 0 | 38 ^**^ | | 0 |
| HP0390 | adhesin-thiol peroxidase | 2 | 5 | | 0 |
| HP0392 | histidine kinase | 0 | 16 | | 0 |
| HP0397 | phosphoglycerate dehydrogenase | 0 | 2 | | 0 |
| HP0410 | putative neuraminyllactose-binding hemagglutinin homolog | 0 | 14 | | 0 |
| HP0415 | conserved hypothetical integral membrane protein | 0 | 3 | | 0 |
| HP0417 | methionyl-tRNA synthetase | 0 | 8 | | 0 |
| HP0434 | hypothetical protein | 0 | 2 | | 1 |
| HP0448 | hypothetical protein | 0 | 0 | | 2 |
| HP0485 | catalase-like protein | 3 | 0 | | 1 |
| HP0486 | hypothetical protein | 0 | 3 | | 0 |
| HP0516 | heat shock protein | 0 | 2 | | 0 |
| HP0550 | transcription termination factor Rho | 0 | 2 | | 0 |
| HP0558 | beta ketoacyl-acyl carrier protein synthase II | 0 | 4 | | 0 |
| HP0561 | 3-ketoacyl-acyl carrier protein reductase | 2 | 6 | | 1 |
| HP0570 | aminopeptidase | 3 | 2 | | 1 |
| HP0588 | ferrodoxin-like protein | 0 | 2 | | 1 |
| HP0589 | ferredoxin oxidoreductase, alpha subunit | 1 | 8 | | 6 |
| HP0590 | ferredoxin oxidoreductase, beta subunit | 0 | 3 | | 0 |
| HP0591 | ferredoxin oxidoreductase, gamma subunit | 1 | 3 | | 0 |
| HP0596 | lipoprotein, putative | 0 | 3 | | 0 |
| HP0599 | hemolysin secretion protein precursor | 0 | 18 | | 0 |
| HP0605 | hypothetical protein | 0 | 16 | | 0 |
| HP0606 | membrane fusion protein | 3 | 4 | | 4 |
| HP0607 | acriflavine resistance protein | 2 | 4 | | 0 |
| HP0629 | hypothetical protein | 1 | 1 | | 0 |
| HP0632 | quinone-reactive Ni-Fe hydrogenase, large subunit | 0 | 3 | | 0 |
| HP0649 | aspartate ammonia-lyase | 0 | 3 | | 0 |
| HP0657 | processing protease | 3 | 0 | | 0 |
| HP0690 | acetyl coenzyme A acetyltransferase | 0 | 2 | | 0 |
| HP0695 | hydantoin utilization protein A | 7 | 17 | | 4 |
| HP0696 | N-methylhydantoinase | 4 | 13 | | 3 |
| HP0697 | hypothetical protein | 0 | 4 | | 0 |
| HP0786 | preprotein translocase subunit | 0 | 3 | | 0 |
| HP0797 | flagellar sheath adhesin | 0 | 3 | | 1 |
| HP0825 | thioredoxin reductase | 0 | 2 | | 0 |
| HP0829 | inosine-5'-monophosphate dehydrogenase | 3 | 0 | | 1 |
| HP0859 | ADP-L-glycero-D-mannoheptose-6-epimerase | 0 | 2 | | 0 |
| HP0875 | catalase | 17 | 0 | | 15 |
| HP0883 | Holliday junction DNA helicase | 0 | 31 | | 0 |
| HP0887 | vacuolating cytotoxin | 7 | 30 | | 10 |
| HP0913 | outer membrane protein 21 | 0 | 1 | | 1 |
| HP0978 | cell division protein | 0 | 2 | | 0 |
| HP1019 | serine protease | 1 | 8 | | 5 |
| HP1069 | cell division protein | 0 | 5 | | 0 |
| HP1118 | gamma-glutamyltranspeptidase | 4 | 0 | | 3 |
| HP1125 | peptidoglycan associated lipoprotein precursor | 0 | 3 | | 0 |
| HP1126 | colicin tolerance-like protein | 1 | 1 | | 1 |
| HP1131 | ATP synthase F1, subunit epsilon | 0 | 2 | | 0 |
| HP1132 | ATP synthase F1, subunit beta | 1 | 24 | | 5 |
| HP1134 | ATP synthase F1, subunit alpha | 1 | 36 | | 4 |
| HP1135 | ATP synthase F1, subunit delta | 0 | 7 | | 0 |
| HP1147 | ribosomal protein L19 | 0 | 4 | | 0 |
| HP1152 | signal recognition particle protein | 0 | 2 | | 0 |
| HP1172 | glutamine ABC transporter, periplasmic glutamine-binding protein | 0 | 20 | | 0 |
| HP1195 | translation elongation factor EF-G | 0 | 7 | | 0 |
| HP1198 | DNA-directed RNA polymerase, beta and beta' subunit | 1 | 2 | | 0 |
| HP1200 | ribosomal protein L10 | 0 | 1 | | 1 |
| HP1205 | translation elongation factor EF-Tu | 6 | 73 | | 3 |
| HP1241 | alanyl-tRNA synthetase | 0 | 8 | | 0 |
| HP1243 | outer membrane protein 28 | 1 | 31 | | 1 |
| HP1245 | single-strand DNA-binding protein | 0 | 125 ^***^ | | 0 |
| HP1279 | anthranilate isomerase | 0 | 30 | | 0 |
| HP1286 | conserved hypothetical secreted protein | 3 | 1 | | 2 |
| HP1293 | DNA-directed RNA polymerase, alpha subunit | 0 | 5 | | 0 |
| HP1301 | ribosomal protein L15 | 4 | 0 | | 2 |
| HP1302 | ribosomal protein S5 | 0 | 3 | | 0 |
| HP1304 | ribosomal protein L6 | 2 | 5 | | 1 |
| HP1310 | ribosomal protein S17 | 0 | 2 | | 0 |
| HP1314 | ribosomal protein L22 | 1 | 1 | | 0 |
| HP1318 | ribosomal protein L4 | 9 | 1 | | 4 |
| HP1319 | ribosomal protein L3 | 0 | 6 | | 1 |
| HP1320 | ribosomal protein S10 | 0 | 4 | | 0 |
| HP1325 | fumarase | 0 | 2 | | 0 |
| HP1345 | phosphoglycerate kinase | 0 | 9 | | 0 |
| HP1350 | protease | 23 | 29 | | 71 ^***^ |
| HP1375 | UDP-N-acetylglucosamine acyltransferase | 0 | 5 | | 0 |
| HP1385 | fructose-1,6-bisphosphatase | 0 | 2 | | 0 |
| HP1399 | arginase | 1 | 1 | | 0 |
| HP1428 | conserved hypothetical protein | 1 | 1 | | 0 |
| HP1454 | hypothetical protein | 2 | 0 | | 0 |
| HP1457 | hypothetical protein | 0 | 2 | | 0 |
| HP1460 | DNA polymerase III alpha-subunit | 2 | 1 | | 0 |
| HP1488 | conserved hypothetical secreted protein | 1 | 6 | | 0 |
| HP1496 | general stress protein | 0 | 3 | | 0 |
| HP1542 | hypothetical protein | 0 | 4 | | 0 |
| HP1554 | ribosomal protein S2 | 0 | 2 | | 0 |
| HP1563 | alkyl hydroperoxide reductase | 19 | 18 | | 15 |
| HP1588 | conserved hypothetical protein | 1 | 21 | | 2 |
|  | Total Spectral Counts | 208 | 1428 | | 237 |
| ^a^ Based on the *H. pylori* 26695 genome annotation | | | |  | |
| ^b^ CagH-HA and CagL-HA were affinity purified from strains expressing these proteins using an anti-HA antibody, and a WT strain was processed in parallel as a control. The Table shows numbers of spectral counts observed by MudPIT analysis for each identified protein. | | | |  | |
| ** p<0.01; *** p<0.001 when compared to WT control, according to the G-test likelihood ratio, post-spectral count normalization. | | | |  | |
