# Supplementary material for: Helicobacter pylori Exploits a Unique Repertoire of Type IV Secretion System Components for Pilus Assembly at the Bacteria-Host Cell Interface
Source: PLoS Pathog. 2011 Sep 1;7(9):e1002237. doi: 10.1371/journal.ppat.1002237 (PMC3164655; doi:10.1371/journal.ppat.1002237)
Supplement: Table S3 — Cag proteins that co-purify with CagL in the absence of CagH or CagI. (DOC) [file ppat.1002237.s003.doc]

**Table S3. Cag proteins that co-purify with CagL in the absence of CagH or CagI**

| **Gene Number ^a^** | **Protein** | **WT ^b^** | **Δ*cagH* ^b^** | **Δ*cagI* ^b^** |  |
| --- | --- | --- | --- | --- | --- |
| HP0539 | CagL | 43 | 25 ^*^ | 22 |  |
| HP0540 | CagI | 60 | 0 ^***^ | 0 ^***^ |  |
| HP0541 | CagH | 22 | 0 ^***^ | 3 ^**^ |  |
| HP0530 | CagV | 16 | 13 | 11 |  |
| HP0544 | CagE | 4 | 0 | 2 |  |
| HP0547 | CagA | 82 | 74 | 42 ^*^ |  |
| HP0524 | Cag5 | 6 | 9 | 3 |  |
| HP0529 | CagW | 2 | 1 | 6 |  |
| HP0526 | CagZ | 0 | 1 | 2 |  |
| HP0543 | CagF | 1 | 2 | 1 |  |
| HP0528 | CagX | 2 | 0 | 1 |  |
| HP0527 | CagY | 1 | 3 | 0 |  |
| Total Spectral Counts | | 8665 | 8768 | 6667 |  |
| ^a^ Based on the *H. pylori* 26695 genome annotation | | | |  |  |
| ^b^ CagL was affinity purified from the WT strain, Δ*cagH* and Δ*cagI* mutants (each cultured for 24 h) using anti-CagL polyclonal antiserum. The Table shows numbers of spectral counts observed by MudPIT analysis for each identified Cag protein. | | | | | |
| *p<0.05; ** p<0.01; *** p<0.001 when comparing each of the mutant strains with WT, according to the G-test likelihood ratio, post-spectral count normalization. | | | | | |
