# Supplementary material for: Helicobacter pylori Exploits a Unique Repertoire of Type IV Secretion System Components for Pilus Assembly at the Bacteria-Host Cell Interface
Source: PLoS Pathog. 2011 Sep 1;7(9):e1002237. doi: 10.1371/journal.ppat.1002237 (PMC3164655; doi:10.1371/journal.ppat.1002237)
Supplement: Table S4 — Co-purification of CagH, CagI, and CagL from H. pylori attached to gastric epithelial cells. (DOC) [file ppat.1002237.s004.doc]

**Table S4. Co-purification of CagH, CagI, and CagL from *H. pylori* attached to gastric epithelial cells**

|  |  | **α-HA^b^** | |
| --- | --- | --- | --- |
| **Gene Number ^a^** | **Protein** | **WT** | **CagH-HA** |
| HP0539 | CagL | 0 | 24 ^***^ |
| HP0540 | CagI | 0 | 25 ^***^ |
| HP0541 | CagH | 0 | 20 ^**^ |
| HP0527 | CagY | 0 | 2 |
| Total Spectral Counts | | 35 | 220 |
| ^a^ Based on the *H. pylori* 26695 genome annotation | | | |
| ^b^ An *H. pylori* strain expressing CagH-HA and a WT strain were each co-cultured with AGS cells for 5 h. CagH-HA was affinity purified using an anti-HA antibody, and the WT co-culture sample was processed in parallel as a control. The Table shows numbers of spectral counts observed by MudPIT analysis for each identified Cag protein. | | | |
| ** p<0.01; *** p<0.001 when compared to WT control, according to the G-test likelihood ratio, post-spectral count normalization. | | | |
